# Plankton biogeography in the North Atlantic Ocean and its adjacent seas: Species assemblages and environmental signatures

**DOI:** 10.1002/ece3.7406

**Published:** 2021-03-24

**Authors:** Loïck Kléparski, Grégory Beaugrand, Martin Edwards

**Affiliations:** ^1^ CNRS UMR 8187 – LOG – Laboratoire d’Océanologie et de Géosciences Univ. Littoral Côte d’Opale, Univ. Lille Wimereux France; ^2^ Continuous Plankton Recorder (CPR) Survey The Marine Biological Association Plymouth UK; ^3^ Marine Institute Plymouth University Plymouth UK

**Keywords:** biogeography, coenoclines, environmental signature, macroecology, North Atlantic Ocean, plankton, taxonomic assemblages

## Abstract

Plankton biodiversity is a key component of marine pelagic ecosystems. They are at the base of the food web, control the productivity of marine ecosystems, and provide many provisioning and regulating ecological services. It is therefore important to understand how plankton are organized in both space and time. Here, we use data of varying taxonomic resolution, collected by the Continuous Plankton Recorder (CPR) survey, to map phytoplankton and zooplankton biodiversity in the North Atlantic and its adjacent seas. We then decompose biodiversity into 24 species assemblages and investigate their spatial distribution using ecological units and ecoregions recently proposed. Finally, we propose a descriptive method, which we call the environmental chromatogram, to characterize the environmental signature of each plankton assemblage. The method is based on a graphic that identifies where species of an assemblage aggregate along an environmental gradient composed of multiple ecological dimensions. The decomposition of the biodiversity into species assemblages allows us to show (a) that most marine regions of the North Atlantic are composed of coenoclines (i.e., gradients of biocoenoses or communities) and (b) that the overlapping spatial distribution of assemblages is the result of their environmental signatures. It follows that neither the ecoregions nor the ecological units identified in the North Atlantic are characterized by a unique assemblage but instead by a mosaic of assemblages that overlap in many places.

## Graphical abstract

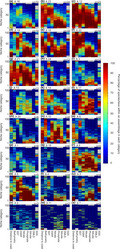

## 1 INTRODUCTION

Plankton are a key component of marine pelagic ecosystems controlling their productivity (Edwards et al., 2013). Phytoplankton produce by photosynthesis almost half of the oxygen at the global scale (Behrenfeld, 2014). They create endosomatic energy that is progressively channeled through the whole marine food web. Zooplankton ensure the transfer of this energy between phytoplankton and higher trophic levels such as fish. Any changes in the abundance and composition of plankton affect higher trophic levels (Edwards et al., 2013; Luczak et al., 2011). Plankton also control a part of carbon exportation in the North Atlantic by a process termed the biological carbon pump (Brun et al., 2019). Plankton are also good indicators of climate change impacts because they are in general not commercially exploited and have a relatively short life cycle.

Understanding the influence of climate change on plankton necessitates having a good understanding of its spatial distribution and its natural annual variations (Reid & Edwards, 2001). However, large‐scale plankton‐monitoring programs are rare (Richardson & Poloczanska, 2008). In the North Atlantic and some of its adjacent seas, plankton have been investigated by the Continuous Plankton Recorder (CPR) survey on a monthly basis since 1946 (Reid et al., 2003). Recently, the survey has been implicated in the European research project ATLANTOS, gathering 18 countries with the aims of bringing together all the existing Atlantic Ocean observing activities into a more integrated wide observation system (AtlantOS, 2019).

As part of the ATLANTOS project, a new partition of the North Atlantic Ocean and adjacent seas has been proposed (Beaugrand, Edwards, et al., 2019). This new partition divides the North Atlantic into 13 ecological units and 40 ecoregions (see Figures S1 and S2). An ecological unit is a group of observations with a homogeneous environmental regime, similar biodiversity and seasonal variability. It can be subsequently divided into ecoregions that are a group of interconnected observations (Beaugrand, Edwards, et al., 2019). The partition was primarily based on spatiotemporal fluctuations in plankton biodiversity but also physical data (e.g., bathymetry, SST, mean surface current). This new partition was built at a relatively fine spatial resolution (i.e., 0.5° latitude × 0.5° longitude) in contrast to previous global studies (Longhurst, 1998) and was the result of the analysis of six key plankton groups (dinoflagellates, diatoms, small and large copepods, small and large zooplankton other than copepods). However, the study did not decompose the biodiversity into species groups, being based exclusively on spatial and temporal (i.e., 2‐month time periods) changes in taxonomic richness.

The main objective of this study was to decompose the biodiversity into species assemblages and to relate them with the partition proposed by Beaugrand, Edwards, et al. (2019). Such a work is needed to provide an information on the biotic composition of the ecological units and ecoregions of the North Atlantic and its adjacent seas. Although a study has identified assemblages of calanoid copepods (108 species or taxa), no joint analyses have been performed on both phytoplankton and zooplankton collected from the CPR survey (Beaugrand et al., 2002). We identified taxonomic assemblages using all taxa (species, genera, or higher taxonomic resolution) recorded by the CPR survey. All phytoplankton and zooplankton were considered and combined together to be associated into groups of taxa. First, we gridded spatially the abundance of all taxa for each 2‐month period (1948–2016). We assumed that spatial variance was much more pronounced than temporal (i.e., year‐to‐year to decadal) variance. To minimize the potential influence of year‐to‐year and decadal variability, we performed data smoothing. Then, we mapped the biodiversity of phytoplankton and zooplankton. We used a cluster analysis to examine the relationships among ~300 plankton taxa that had a level of abundance sufficiently high to be analyzed. Then, we examined the spatial distribution and annual changes in each assemblage. We used nine environmental parameters to characterize the environmental signature of each assemblage (i.e., a combination of environmental variable that characterizes a species group) using a procedure that leads to the display of what we propose to call an environmental chromatogram (i.e., a graphic that identifies where species of an assemblage aggregate along an environmental gradient composed of multiple ecological dimensions). We prefer the term environmental signature (i.e., the environmental regime where species aggregation within an assemblage is highest) instead of the term ecological niche because the latter is usually restricted to the species level (Chase & Leibold, 2003). Finally, we examined the composition of each ecoregion and ecological unit (sensu Beaugrand, Edwards, et al., 2019) in terms of assemblage.

## 2 MATERIALS AND METHODS

### 2.1 Physical data

We used a set of nine physical variables: sea surface temperatures (SST), bathymetry, monthly mean downward solar radiation flux at surface (DSRF), macronutrients (nitrate, phosphate, and silicate), distance to nearest coastlines, density mixed layer depth (DMLD), and sea surface salinity (SSS). Information on data sources can be found in Appendix 1.

### 2.2 Biological data, the CPR survey

Biological data originated from the Continuous Plankton Recorder (CPR) survey. It is a long‐term plankton‐monitoring program currently operated by the Marine Biological association of Plymouth. The CPR is the longest and most extensive program of that kind in the world. The machine is a high‐speed plankton recorder towed behind voluntary merchant ships, called “ships of opportunity,” that filters phytoplankton and zooplankton at a depth of ~7 m (Hays & Warner, 1993). The taxonomic resolution of the data used in this study is shown in Table S1. More information about the CPR survey (advantages and limitations) can be found in Text S1 and in Section 4.1 of the Discussion. Information on data sources can be found in Appendix 1.

### 2.3 Mathematical analyses

Six main analyses, all coded in MATLAB, were performed in this study (Figure S3).

#### 2.3.1 Analysis 1. Spatial regularization

Spatial sampling by the CPR survey is heterogeneous. Therefore, we first created a spatial grid for every plankton species/taxa sampled by the CPR survey for every two‐month period using data collected between 1948 and 2016. The spatial grids were identical to the ones used in Beaugrand, Edwards, et al. (2019). For each geographical cell of 0.5° of latitude and 0.5° of longitude from 80.5°W to 9.5°E and from 40.5°N to 65.5°N, we calculated average abundance values of each plankton taxa for every two‐month period based on the period 1948–2016. The procedure led to a three‐dimensional matrix of 304 taxa × 9,231 geographical cells (latitudes × longitudes) × 6 two‐month periods.

#### 2.3.2 Analysis 2. Spatial smoothing of the gridded data

Abundance data can be highly variable from a geographical cell to another which can be in part attributed to the CPR sampling (e.g., variable seawater filtered by the machine, ship speed) and year‐to‐year variability (Jonas et al., 2004). We therefore smoothed spatially all two‐month abundance grids for each species/taxa. Smoothing was performed by applying a c‐order spatial simple moving average:

$$y_{i,j}=\frac{1}{{\left(2c+1\right)}^{2}}\sum_{s=i‐c}^{i+c}\sum_{t=j‐c}^{j+c}x_{s,t}$$

with 1+c≤i≤k‐c and 1+c≤j≤l‐c, *k* the number of latitudes (*k* = 51) and *l* the number of longitudes (*l* = 181).

*y_i,j_* is the smoothed abundance value of a species/taxa at the geographical cell corresponding to latitude *i* and longitude *j* and *x* is the original abundance value for a species/taxa. Threshold selection *c* depends upon the size of geographical cells (here a cell is 0.5° latitude × 0.5° longitude), the noise inherent to the data, and the type and the location of spatial structures. By trial and error, we fixed *c* to 2 as a compromise between noise reduction and potential numerical artifacts. Many values can be missing in some areas, and we fixed to 20 the maximum number of missing values allowed to have an estimation.

#### 2.3.3 Analysis 3. Spatial distribution of the taxonomic richness

**FIGURE 1:**
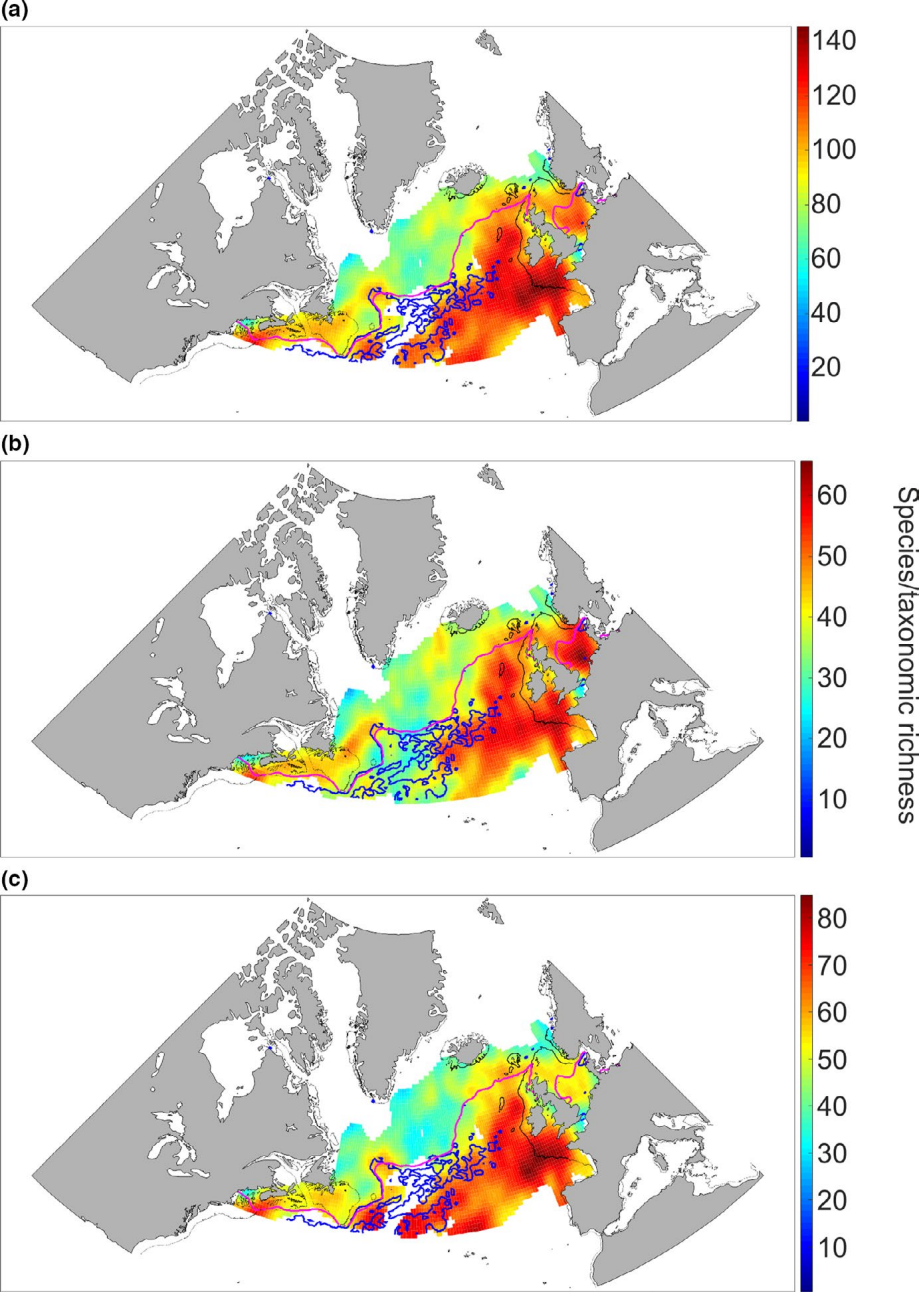
Spatial distribution of total taxonomic richness (a), phytoplankton taxonomic richness (b), and zooplankton taxonomic richness (c) sampled by the CPR survey in the North Atlantic Ocean. The magenta line corresponds to isotherm 10°C. The blue lines correspond to current velocities from 0.5 to 2 m/s. The black lines denote isobath 200 m. We caution that phytoplankton and zooplankton biodiversity maps were based on taxa identified at the species (72.5% for phytoplankton and 47.1% for zooplankton), genus (26.2% and 23.9%), and higher taxonomic (1.3% and 29%) resolutions

The spatial distribution of taxonomic richness was mapped using the 3‐dimensional matrix resulting from Analyses 1 and 2. For this analysis, abundance was converted into presence–absence using a threshold of 0 (i.e., abundance > 0 means an occurrence). The use of other thresholds did not affect our conclusions. We then summed all presence for phytoplankton and/or zooplankton species or taxa and smoothed the resulting matrix by applying a first‐order spatial triple moving average (*c* = 1) to obtain a map of the spatial distribution of taxonomic richness. The analysis was performed for total (i.e., phytoplankton and zooplankton) taxonomic richness, phytoplankton taxonomic richness, and zooplankton taxonomic richness. Among the 304 taxa we used, 149 were phytoplankton, 155 were zooplankton; ~60% of plankton taxa were identified at the species level (Figure 1 and Table S1).

#### 2.3.4 Analysis 4. Identification of taxonomic assemblages

We then decomposed the biodiversity into taxonomic assemblages. We first calculated a squared distance matrix (taxa × taxa) using the Hellinger distance coefficient, which is robust to a high number of double zeros (Legendre & Legendre, 1998):

$$\text{Distance}\left(\text{species}\;1,\text{species}\;2\right)=\sqrt{\sum_{j=1}^{p}{\left[\sqrt{\frac{y_{1j}}{y_{1+}}}‐\sqrt{\frac{y_{2j}}{y_{2+}}}\right]}^{2}}$$

with $y_{1j}$ the abundance of the species/taxa 1 in geographical cell *j*, $y_{1+}$, the total abundance of species/taxa 1 across all geographical cells, $y_{2j}$ the abundance of species/taxa 2 in geographical cell *j*, $y_{2+}$ the total abundance of species/taxa 2 across all geographical cells.

The distance coefficient was calculated between species/taxa on the basis of their patterns of abundance in space (i.e., all geographical cells covering the Atlantic Ocean) and time (i.e., two‐month period).

Prior to the use of Hellinger's distance coefficient, abundance data were transformed using the function $\log_{10}\left(x+1\right)$, a procedure frequently applied to the CPR data that also clearly limit the Euclidean's distance paradox.

**FIGURE 2:**
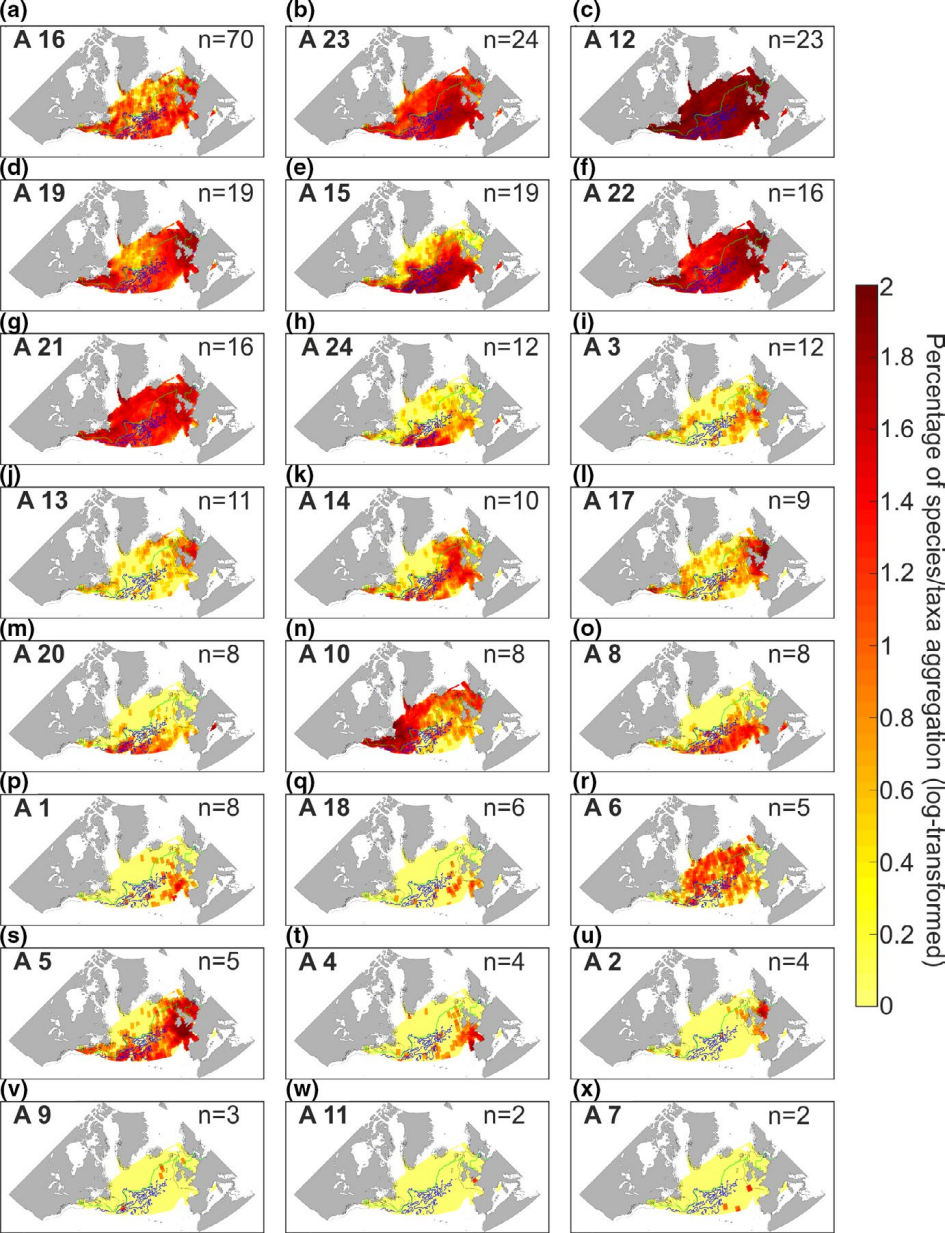
Mean spatial distribution of taxonomic assemblages identified by the cluster analysis (see Figure S4 and Table S1) and based on an average of six 2‐month period (1948–2016). On the top right of each panel, the number of species/taxa (n) in the assemblage is indicated. The assemblage number, corresponding to the numbers in Figure S4, is displayed in bold on the top left. The green line corresponds to isotherm 10°C. The blue lines correspond to current velocities from 0.5 to 2 m/s. The black lines denote the isobath 200 m. Panels are classified from a to x by decreasing taxonomic richness

**FIGURE 3:**
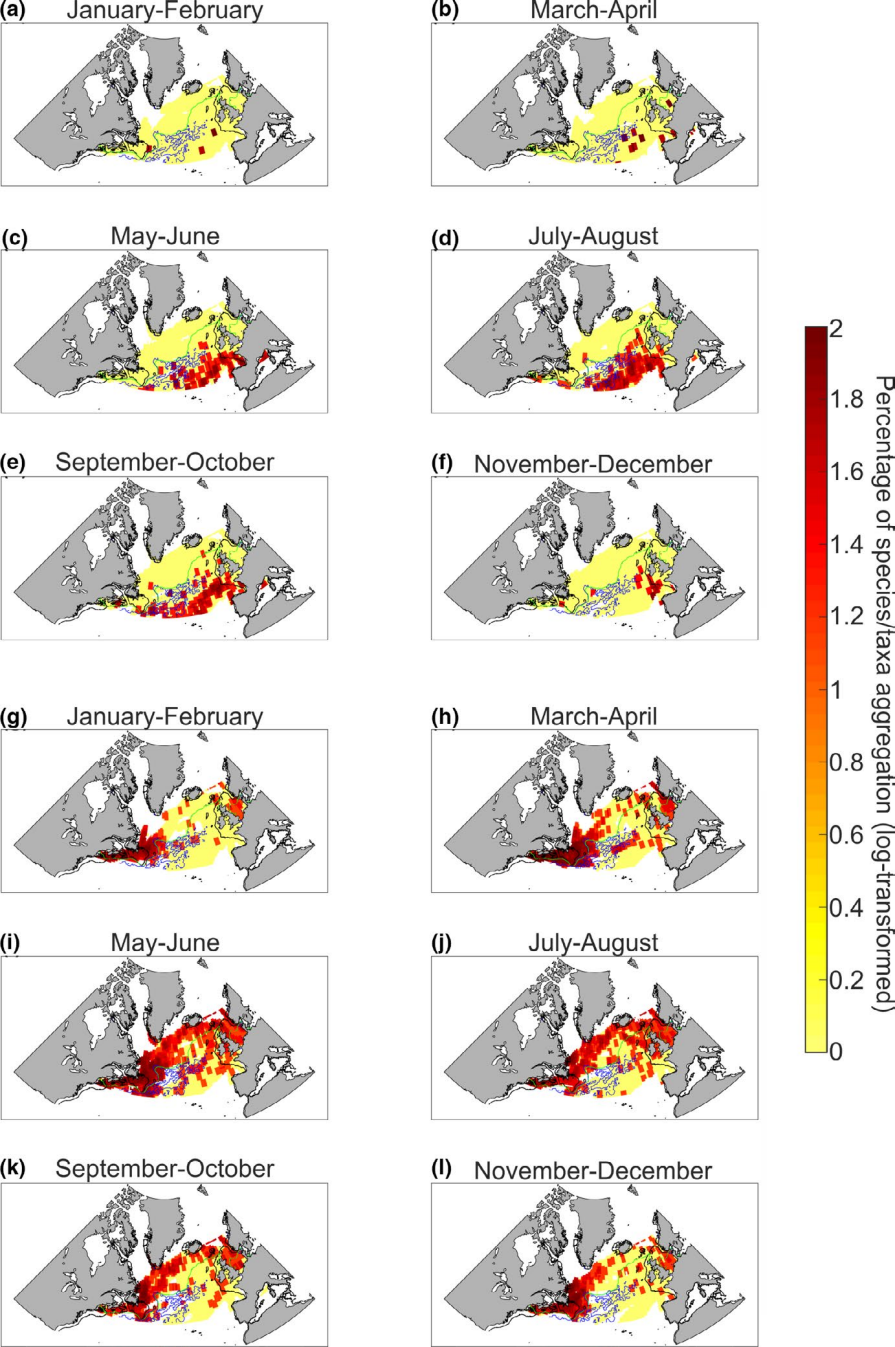
Seasonal changes in the spatial distribution of Assemblage 8 (panels a–f) and 10 (panels g–l). The green line corresponds to isotherm 10°C. The blue lines correspond to current velocities from 0.5 to 2 m/s. The black lines denote the isobath 200 m

A cluster analysis was subsequently applied using Ward's minimum variance method (Legendre & Legendre, 1998). The resulting dendrogram is in Figure S4. Spatial distribution of all assemblages was subsequently mapped by applying the same procedure to represent the spatial distribution of taxonomic richness (Analysis 3) but at the assemblage level. For each assemblage, we mapped the percentage of species/taxa aggregation (i.e., percentage of co‐occurring taxa of a given taxonomic assemblage in a cell) by averaging the maps built for each 2‐month period (1948–2016; Figure 2). The procedure was also applied for each two‐month period to examine seasonal changes in each taxonomic assemblage (Figure 3).

Taxonomic composition of each assemblage was characterized by the use of pie charts in two ways:

- First, we determined the number of species/taxa belonging to six categories: (i) diatoms, (ii) dinoflagellates, (iii) other phytoplankton, (iv) large copepods, (v) small copepods, and (vi) other zooplankton (Figure S5).
- Second, we determined the number of taxa identified at (i) a species level and (ii) a higher taxonomic resolution (Figure S6).

#### 2.3.5 Analysis 5. Relationships between taxonomic assemblages and both ecoregions and ecological units identified by Beaugrand, Edwards, et al. (2019)

**FIGURE 4:**
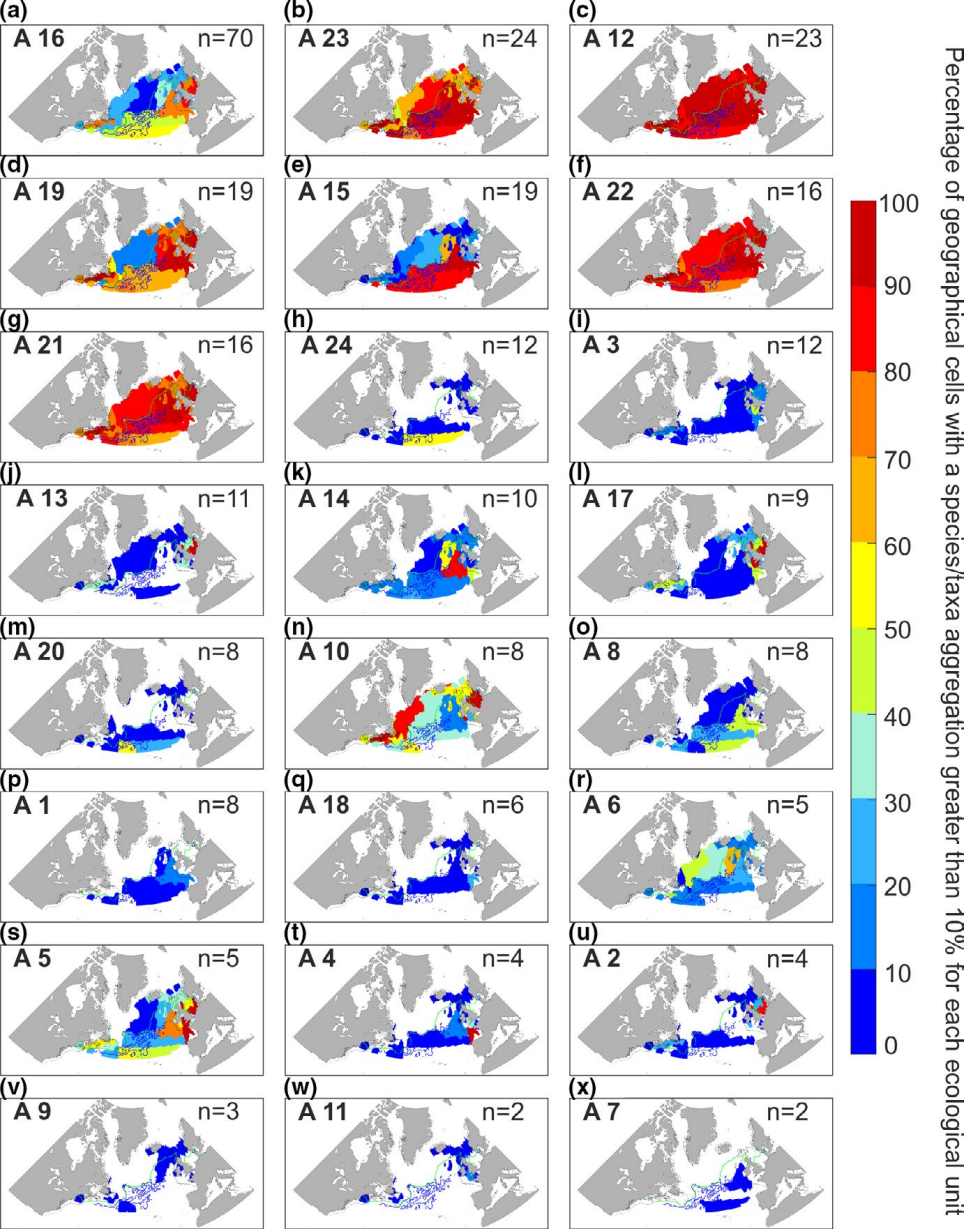
Percentage of taxonomic aggregation (i.e., number of species or taxa of the same assemblage) greater than 10% in each ecological unit as defined by Beaugrand, Edwards, et al., (2019)). Here, the percentage of species/taxa aggregation is used to identify an assemblage characteristic of an ecological unit. On the top right of each panel, the number of species/taxa (n) in the assemblage is indicated. The assemblage number, corresponding to the numbers in Figure S4 and in Figure 2, is displayed in bold on the top left. The green line corresponds to isotherm 10°C. The blue lines correspond to current velocities from 0.5 to 2 m/s. The black lines denote isobath 200 m. Panels are sorted from a to x by decreasing taxonomic richness

From Analysis 4, we calculated the percentage of geographical cells with a percentage of taxonomic aggregation (i.e., percentage of co‐occurring taxa of a given taxonomic assemblage in a cell) higher than 10% (Figure 4 and Figure S7) and 50% (Figures S8 and S9) in all ecoregions (Figures S7 and S8) and ecological units (Figure 4 and Figure S9) sensu Beaugrand, Edwards, et al. (2019). This analysis was performed to determine the taxonomic assemblage composition of each ecoregion and ecological unit.

#### 2.3.6 Analysis 6. Estimation of the environmental signature of each taxonomic assemblage

We used a set of nine environmental variables (see Physical data section) covering the whole North Atlantic Ocean and adjacent seas in space and time to characterize the environmental signature of each assemblage by means of what we call an environmental chromatogram, that is, a graphic that identifies where species of an assemblage aggregate along an environmental gradient composed of multiple ecological dimensions. First, the value of each environmental variable was interpolated using the grid we used for plankton data. With the exception of bathymetry and distance to coast, all data were linearly interpolated for each two‐month period. We therefore obtained a matrix 9,231 geographical cells × 6 by two‐month period for each environmental variable; note that for bathymetry and distance to coast, the same values were repeated for each two‐month period. By this way, it was possible to relate the abundance of each species/taxa with any environmental variable in space and time. All values of each environmental variable were then standardized between 0 (i.e., lowest value) and 1 (highest value).

**FIGURE 5:**
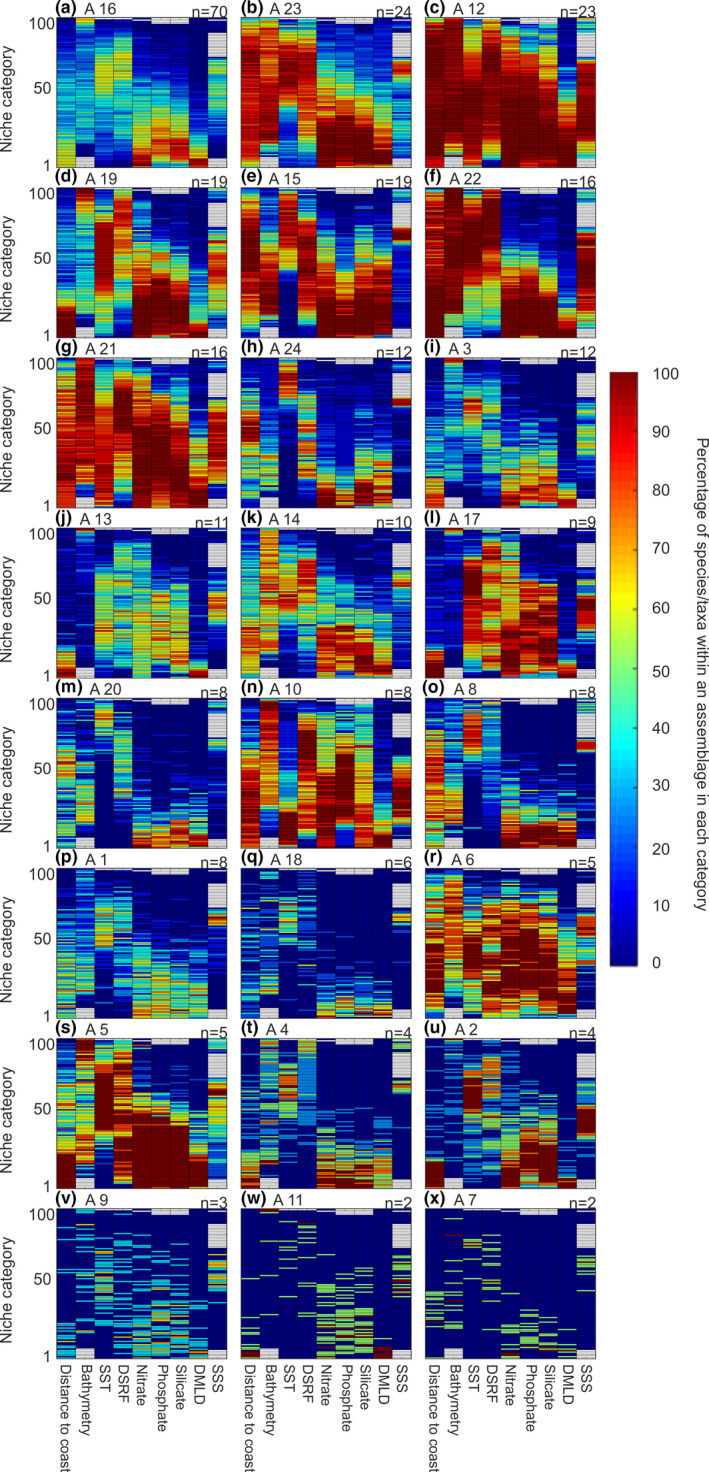
Environmental signature of the 24 species/taxa for all 24 assemblages. For each figure, from a to x, column corresponds to all the values take by an environmental variable (e.g., distance to coast or silicate). For each variables (column), all values were divided into 100 categories standardized between 0 and 1, bottom categories (0) corresponding to the smallest values taken by an environmental variable. Color indices denote the percentage of species/taxa of an assemblage found into a category. Red color indicates that the majority of the species/taxa composing the assemblages are found in these environmental categories. Blue color indicates that no species/taxa or a few were found in these environmental categories. Panels are classified from a to x by decreasing taxonomic richness. The number at the top left of each panel corresponds to the assemblage number (see Figure 2 and Figure S4), and the number at the top right (n) indicates the taxonomic richness of each assemblage

We then divided all environmental values between 0 (lowest values) and 1 (highest values) into 100 categories and calculated the abundance of each species/taxa that corresponded to each environmental category between 0 and 1. The choice of the categories (100) resulted from a compromise between the resolution of the chromatogram and the number of observed environmental values (here 9,231 × 6 = 55,386 if we include missing values). Because the number of values was high, we chose 100 categories to improve the resolution. The standardization of each environmental dimension between 0 and 1 allowed (a) their representation inside a two‐dimensional space and (b) the subsequent characterization of the environmental signature of each assemblage (Figure 5); we propose to call this graphic an environmental chromatogram. The environmental signature of each assemblage was the result of the ecological niche of all species in that assemblage.

We also characterized the environmental signature of all taxonomic assemblages by considering phytoplankton and zooplankton separately (Figure S10). This comparison was made to check whether signatures were identical. By this way, we checked the homogeneity of phytoplankton and zooplankton signatures among each species assemblage. We then compared patterns of environmental signature for phytoplankton and zooplankton by means of a correlation analysis. We used a Spearman correlation coefficient tested by a Monte Carlo test using 10,000 permutations to estimate the probability (Jackson & Somers, 1989).

## 3 RESULTS

Figure 1a shows the spatial distribution of taxonomic richness in the North Atlantic Ocean and its adjacent seas based on 304 phytoplankton and zooplankton species/taxa. A strong gradient was observed between the northern and the southern part of the oceanic basin, with an increasing taxonomic richness toward the equator (we used data with different degrees of taxonomic resolution, see Table S1). A second gradient was also observed between the western and the eastern sides of the Atlantic Ocean, with an increasing taxonomic richness eastward. Biodiversity was greatest south of the 10°C isotherm (magenta line in Figure 1) with taxonomic richness varying between 100 and 140 taxa in the south and between 60 and 80 in the north. Higher taxonomic richness occurred in areas characterized by warmer temperatures (e.g., West European Basin) and high current velocity (e.g., the Gulf‐Stream). Similar patterns in taxonomic richness were observed for phytoplankton (Figure 1b) and zooplankton (Figure 1c) although maximal taxonomic richness was observed in the North Sea for phytoplankton and in the West European Basin for zooplankton. Zooplanktonic taxonomic richness was higher than phytoplanktonic taxonomic richness in the Gulf‐Stream Extension.

Plankton biodiversity was then decomposed into 24 plankton assemblages (Figure 2) by means of a cluster analysis (see Figure S4). We chose a cutoff level of 1.7 in the dendrogram to select a maximum of groups. When thresholds were too high (i.e., >1.7), groups remained too spatially and/or temporally heterogeneous, and when thresholds were too small, we had too many isolated species. Five assemblages (Assemblages 16, 23, 12, 19, 22, and 21; Figure 2a–d,f–g) occurred nearly everywhere in the studied area; they were therefore eurygraphic. All the remaining assemblages were located south of the 10°C isotherm, except Assemblage 10 (Figure 2n) observed in the northern part of our studied area and Assemblage 6 mainly detected nearly everywhere in the open ocean (Figure 2r). Among the eighteen southern assemblages, three (Assemblages 1, 18, and 4; Figure 2p,q,t) occurred in the West European Basin including the Bay of Biscay and four (Assemblages 3, 13, 17, and 2; Figure 2i,j,l,u) were mainly located over continental shelves. Assemblage 5 (Figure 2s) was located south of the Oceanic Polar Front (OPF) sensu Dietrich 1964 (Dietrich, 1964), over continental shelves and in open ocean. The remaining assemblages (i.e., Assemblages 15, 24, 14, 20, 8, 9, 11, and 7) mainly occurred south of the 10°C isotherm in the open ocean (Figure 2e,h,k,m,o,v,w,x).

Annual changes were examined for all assemblages. We only show such changes for Assemblages 8 (Figure 3a–f) and 10 (Figure 3g–l) as examples. Both assemblages exhibited strong seasonal variations in taxonomic aggregation throughout the year. Assemblage 8 was virtually absent from November to April in the surface. From May to October, there was a substantial increase in taxonomic aggregation south of the OPF (see the 10°C isotherm in Figure 3). Assemblage 10 (Figure 3g–l) was observed over continental shelves (American and European) for all 2‐month periods. From March to October, this assemblage spread over the oceanic regions north of the 10°C isotherm in the Subarctic Gyre.

Taxonomic composition and resolution of each assemblage are displayed as pie charts (Figures S5–S6). These last figures show that 18 out of 24 assemblages (75% of the assemblages) were composed of at least 50% of taxa identified at the species level. Each assemblage was indicative of one or more ecological units sensu Beaugrand, Edwards, et al. (2019) (hereafter termed EUs). The name of each unit is indicated in Figure S1. For this analysis, we used a threshold of taxonomic aggregation of 10% (i.e., species or taxa that had a percentage of co‐occurrence higher than 10% in a given geographical cell for a given assemblage). Other thresholds were tried and did not alter substantially our conclusions when they were fixed below 50% (see Figures S8–S9). Four assemblages (Assemblages 23, 12, 22, and 21; Figure 4b,c,f,g) occurred in almost 80% of the geographical cells composing all ecological units. Assemblages 16 (Figure 4a) and 19 (Figure 4d) mainly occurred around the British Isles (e.g., the Cold‐Temperate Neritic and the Cold‐Temperate Shallow Neritic EUs), although they were also detected in oceanic EUs south of the 10°C isotherm (e.g., the Gulf‐Stream Extension, the Northern Sub‐Tropical and the Oceanic Warm‐Temperate EUs [see the nomenclature in Figure S1]). Assemblages 15, 24, and 20 (Figure 4e,h,m) were also present in the last three EUs but mostly in the southern ones, that is, the Gulf‐Stream Extension and the Northern Sub‐Tropical EUs. Assemblages 10 and 6 (Figure 4n,r) were mainly located in northern EUs (e.g., the Polar oceanic EU). Many assemblages (3, 13, 17, 5, and 2 in Figure 4i,j,l,s,u) occurred in EUs covering the continental shelf (e.g., the Cold‐Temperate Shallow Neritic, the Cold‐Temperate Neritic, and the Ocean‐Influenced Cold‐Temperate Neritic EUs) as well as the shelf‐edge for Assemblage 5 (Figure 4s) (i.e., the Diverse and Productive Oceanic and Temperate and the Pseudo‐Oceanic Warm‐Temperate EUs). Remaining assemblages, that is, 14, 8, 1, 18, 4, 9, 11, and 7 (Figure 4k,o,p,q,t,v,w,x), occurred mostly in EUs close to or over the European shelf‐edge, for example, the Diverse and productive Oceanic Temperate EU, the Pseudo‐Oceanic Warm‐Temperate EU, and the Mixed Coastal‐Oceanic Highly‐Seasonally dynamical EU. Therefore, all assemblages were linked to specific EUs of the North Atlantic Ocean and its adjacent seas.

Similar conclusions were reached for ecoregions, although results were more difficult to interpret as ecological units originate from the aggregation of ecoregions (see Figures S8–S9); therefore, the number of ecoregions (40) is higher than the number of ecological units (13) and it is more difficult to find a pattern on a higher number of units.

We characterized the environmental signature of each assemblage by means of what we propose to call an environmental chromatogram (Figure 5). The figure shows the percentage of co‐occurring species/taxa of an assemblage in each environmental category standardized between 0 and 1 (0 being the smallest category and 1 the highest). By this way, we represented nine ecological dimensions (i.e., ecological variables) into two. Each assemblage had its own environmental signature. Assemblages 23, 12, 22, 21, and 6 (Figure 5b,c,f,g,r) occurred in a large number of categories for almost all environmental variables; the species/taxa that compose these assemblages were euryoecious. Other assemblages such as number 16, 24, 3, 20, 1, and 18 (Figure 5a,h,i,m,p,q) were observed in a restricted number of environmental categories; they are more stenoecious.

Then, we compared the environmental signature of phytoplankton and zooplankton species/taxa composing each assemblage in order to check whether signatures were identical. By this way, we verified the homogeneity of phytoplankton and zooplankton signatures among each species assemblage. Expectedly, we found the same environmental signature for phytoplankton and zooplankton of the same assemblage (Figure S10). We also compared the environmental signature of phytoplankton and zooplankton for each assemblage by means of a Spearman correlation coefficient (Table S2 and Figure S10). All correlations were significant (*p* < .01; Table S2). With the exception of four assemblages (1, 4, 9, and 11) that had a correlation lower than 0.5, all assemblages had correlations above 0.5. This result confirmed that the environmental signature of phytoplankton and zooplankton was similar for all assemblages, although the strength of these similarities varied from one assemblage to another.

## 4 DISCUSSION

### 4.1 Potential limitations

Our study has potential limitations related to the nature of the CPR data and to our methodology. First, the CPR is not a perfect sampling mechanism. It underestimates some components of the plankton, for example, large plankton such as fish larvae and delicate gelatinous plankton. Due to the mesh size (~270 µm) of the CPR silk (Jonas et al., 2004), some organisms are only semiquantitatively recorded and abundance of small species is probably underestimated when compared to other water sampling methods. Despite this bias, the proportion of the population that is retained by the CPR silk reflects the major changes in abundance, distribution, and specific composition; that is, the percentage retention is roughly constant within each species even with very small‐celled species (Edwards et al., 2006).

Second, taxonomic identification of plankton has evolved since 1948, which might have affected our results. Nevertheless, on the 304 species/taxa considered in our study, only 27 underwent a change in taxonomic resolution (Batten et al., 2003), which represented less than 9%. Moreover, the taxonomic names recorded in the database have mostly remained unchanged throughout years (Richardson et al., 2006). Some zooplankton species/taxa can also be recorded twice by the CPR taxonomists during the identification laboratory process. The only species recorded in eyecount and traverse analyses in our study was *Centropages chierchiae,* and the two categories were grouped in the same assemblage (i.e., Assemblage 4; Figure 2t). When the same taxon is recorded twice (e.g., Chaetognatha), it is often because it includes different species or developmental stages (e.g., *Euphausiacea* nauplii and *calyptopis*) (Richardson et al., 2006). Because this information was also important for taxonomists and scientists working on the CPR survey, we chose to keep them in our analyses. The choice to keep all categories had no effect on the species assemblages because of the use of the Ward algorithm that minimizes the intragroup variance (Legendre & Legendre, 1998).

Third, sampling by the CPR survey is also restricted to the surface water (~6.5 m in depth; Hays & Warner, 1993), which might affect our perception of how ecosystems are organized. However, a recent study showed that the seasonal and diel patterns in the abundance of *Calanus finmarchicus* at surface were positively correlated with patterns of abundance observed at 100 m (Hélaouët et al., 2016). Despite these limitations, it has been shown that the CPR gives a correct picture of both temporal (i.e., seasonal and diel scale) and spatial (i.e., regional to basin scale) changes in plankton (Batten et al., 2003; Richardson et al., 2006).

It is widely recognized that all plankton sampling systems have their own limitations and nuances and that all underestimate abundance to some degree. However, it is important to note that the CPR survey is the only scientific monitoring program of that kind in the world, with no equivalent existing program. It covers an important timescale from 1948 to present, still active, with a large spatial scale covering the whole North Atlantic Ocean and its adjacent seas such as the Channel, the Celtic Sea, and the North Sea. The CPR is now “the most extensive long‐term survey of marine organisms in the world” (Reid et al., 2003).

Potential limitations also arose because of the methodological choices we made. First, we assumed that the spatial variance was more pronounced than temporal (i.e., year‐to‐year and decadal) variance; we did not consider year‐to‐year to decadal variability. This assumption was needed to cover as fully as possible the North Atlantic Ocean and its adjacent seas. The same assumption was made in Beaugrand, Edwards, et al. (2019) to propose a new partition of the North Atlantic Ocean. The effect of temporal variability was to inflate local spatial variance. To reduce this effect, we spatially smoothed the data (see Analysis 2 in Section 2) prior to conducting other analyses such as the cluster analysis and the identification of environmental signatures. This assumption had no effect on the identification of species assemblages because the environmental signature of assemblages was based upon their spatial and 2‐monthly aggregation for the period 1948–2016. Environmental signatures should be stable at a decadal scale because of niche conservatism (Crisp et al., 2009). Furthermore, the lack of consideration of year‐to‐year to decadal variability did not affect our comparison with the ecological units and ecoregions of Beaugrand, Edwards, et al. (2019) because we considered the same time period. However, the spatial distribution of species assemblages identified in our study is likely to change at a decadal scale with large‐scale hydroclimatic variability (e.g., the Atlantic Multidecadal Oscillation or AMO) (Faillettaz et al., 2019) or global climate change (IPCC et al., 2013).

Second, we considered different levels of taxonomic resolution (e.g., species, genera, order) when we identified the species assemblages. We thought it was important to consider those different levels inside the same analysis because some taxa enable the clear identification of some key ecoregions. For example, Gammaridae, Cumacea, and Mysidacea are mainly found over the continental shelves (Figure 2l). It was also important to consider different developmental stages (e.g., Euphausiacea nauplii and calyptopis) because they are key for ecosystem trophodynamics (Kirby et al., 2008). For example, most eggs and larvae were clustered in Assemblage 19, which was composed of species or taxa mainly abundant south of the Polar Front (Figure 2d). Although the analysis that leads to the identification of species assemblages was not affected by merging different types of entities, this was not so for the maps of biodiversity we present. Our maps therefore display taxonomic richness and not species richness. However, merging different taxonomic entities inside the same analysis did not alter our perception of the spatial difference in biodiversity among regions because patterns observed in this study are close to those observed in other studies that focussed only on calanoid copepods (Beaugrand et al., 2000), *Ceratium*, diatoms (Beaugrand et al., 2010), and phytoplankton (Righetti et al., 2019).

Third to produce abundance maps, we spatially regularized and smoothed the CPR data for each two‐month period. Our procedure gave estimations of gridded abundance (e.g., *Calanus finmarchicus*) similar to that obtained from kriging (Planque et al., 1997), the inverse distance method (Beaugrand et al., 2002), or spatial regularization (Helaouët & Beaugrand, 2007). In addition, our biodiversity maps gave similar biodiversity patterns to those originated from kriging, the inverse square distance method, and spatial regularization using a first‐order jackknife procedure (Beaugrand, 1999; Beaugrand et al., 2000, 2010).

### 4.2 Factors contributing to the large‐scale pelagic biodiversity patterns

We provide a map of plankton taxonomic richness based on both phytoplankton and zooplankton at the North Atlantic basin scale. We caution that phytoplankton and zooplankton biodiversity maps were based on taxa identified at the species (72.5% for phytoplankton and 47.1% for zooplankton), genus (26.2% and 23.9%), and higher taxonomic (1.3% and 29%) levels. Similar maps have been shown in Beaugrand, Edwards, et al. (2019), but the maps we provide here have an improved spatial resolution (2° × 2° vs. 0.5° × 0.5°) due to the implementation of the smoothing algorithm into our procedure of spatial regularization (see Analysis 2 in Section 2).

The examination of the biodiversity maps (Figure 1) has revealed two gradients in taxonomic richness in the North Atlantic: (a) a meridional gradient (south to north) corresponding to the latitudinal biodiversity gradient and (b) a zonal (west to east) gradient. Thus, we observed the greatest biodiversity in the southeastern part of our studied zone and the lowest biodiversity in the Subarctic Gyre. A similar pattern has already been observed for phytoplankton (Righetti et al., 2019) and zooplankton (Beaugrand et al., 2001). Higher taxonomic richness observed south of our studied area coincided with warmer sea surface temperatures and to a lesser extent with oceanic circulation, for example, the Gulf‐Stream and its extension the North Atlantic Current. Some studies have investigated the relationships between temperature and plankton biodiversity using three taxonomic groups and found clear nonlinear relationships between mean and annual variability in temperature and biodiversity (Beaugrand et al., 2010). We have also seen a strong influence of the OPF (identified here by the 10°C isotherm) on biodiversity. These results tend to confirm the influence of the 9–10°C isotherm on plankton as revealed by Beaugrand et al. (2008) using three trophic levels: phytoplankton, zooplankton, and fish. These results also reflect the strong biodiversity difference between the Polar and Westerlies‐Wind biomes sensu Longhurst (1998).

Because biodiversity is low in the subarctic gyre and higher in the south of the OPF, our study suggests that temperature may be an important factor controlling plankton biodiversity in the North Atlantic. During the eighties, Colebrook (Colebrook, 1982, 1985) already suggested a relationship between plankton abundance and temperature. More recently, temperature has been identified as a key driver for both phytoplankton biodiversity and zooplankton biodiversity (Beaugrand et al., 2010). Phytoplankton biodiversity is three times higher in the tropics than in the higher latitudes (Righetti et al., 2019). Such a pattern is related to the Latitudinal Biodiversity Gradient (LBG). The LBG has also been observed for zooplankton (Rombouts et al., 2009).

Other secondary factors may also influence biodiversity patterns at a regional scale. Investigating foraminifera biodiversity in the North Atlantic, Ruddiman (1969) stressed that the biodiversity gradient was virtually erased by the strength of the diverse subtropical North Atlantic gyre. To explain phytoplankton biodiversity patterns in the North Atlantic, Righetti et al. (2019) proposed that phytoplankton biodiversity was influenced by a great species turnover resulting from high seasonal variability in wind stress, turbulence, and light limitation. Margalef (1978) highlighted that there are two important parameters in phytoplankton biology: turbulence that controls sedimentation rates and variance in current velocity that affects β diversity, that is, the differences between local community. An increase in β diversity may also explain why there is a very high taxonomic richness over the Celtic Sea and the Bay of Biscay at the boundary between the continental shelf and the open ocean. The strong spatial variability in the bathymetry in these areas enables the coexistence of oceanic and neritic (meroplankton and holoplankton) species but also pseudo‐oceanic species (i.e., species that occur in the ocean and over the continental shelf but are mainly abundant along the shelf‐edge).

Oceanic circulation has a strong regional or local influence on biodiversity. Many authors have provided evidence that regional biodiversity can be highly influenced by surface currents (Beaugrand et al., 2001; Longhurst, 1998; Ruddiman, 1969; Van der Spoel, 1994). The influence of oceanic circulation is crucial at the Atlantic Basin scale, and the zonal difference in North Atlantic biodiversity is clearly explained by the warmer North Atlantic Current that flows northwards in the eastern part of the North Atlantic (Beaugrand et al., 2002). More locally, the Gulf‐Stream and its northward extension the North Atlantic Current bring more warm‐water species polewards. The OPF (Dietrich, 1964) has a major influence by separating the low biodiversity of the Polar biome (and the subarctic gyre) from regions of higher biodiversity in the Westerlies‐Wind biome (sensu Longhurst, 1998). This influence is stronger on warm‐water species than on cold‐water species. The European shelf‐edge current has also a major influence on the biodiversity of the Bay of Biscay and the western regions of the British Isles in modulating local upwelling and warm‐water advection northwards (Reid et al., 2001). The Labrador Current is characterized by poor biodiversity and the occurrence of a few species such as *Calanus hyperboreus* and *C. glacialis*. In the North Sea, the Flamborough Front has a strong influence on biodiversity by separating stratified waters in the north (i.e., lower taxonomic richness) from mixed waters to the south (i.e., higher taxonomic richness). In the northeastern part of Georges Bank (shelf‐edge at the southeastern part of Newfoundland), Flemish Cape is characterized by a lower taxonomic richness (i.e., ~70 species/taxa; Figure 1a) and the Northwest Corner (Worthington, 1976), which is located to the northeastern part of Newfoundland, has a higher taxonomic richness (i.e. ~100 species/taxa). North of the British Isles, the Faroe Current, and the associated Iceland Faroe Front (Read & Pollard, 1992) limit the spatial location of annual maximum taxonomic richness (Figure 1a). Southwest of the British Isles, maximum taxonomic richness recorded in the Southwest European Basin (west of the Bay of Biscay) may also originate from hydrographic processes. The area closely corresponds with the northward spreading of Intermediate Mediterranean Water (Käse & Zenk, 1996). This water mass has a maximum influence at ~1,000 m depth, but its range extends from 600 to 2,500 m (Käse & Zenk, 1996). Therefore, the influence of hydrographical features resulting from oceanic circulation and in some cases topographic features exerts a strong secondary influence on plankton biodiversity in the North Atlantic by controlling temperature or by controlling more directly plankton advection. Therefore, plankton biodiversity in the North Atlantic Ocean is mainly driven by temperature, oceanic circulation, and bathymetry, which have a more local/regional influence.

### 4.3 Decomposition of plankton biodiversity into assemblages: relationships with North Atlantic ecological units

In a similar study on calanoid copepods, Beaugrand et al. (2002) applied the IndVal method (Dufrêne & Legendre, 1997) in order to group species into indicator assemblages. In our study, the IndVal would not work because the ecoregions used to calculate the indicator values are too heterogeneous (i.e., the size of the ecoregions varied strongly), which would inflate the number of indicator values in smaller ecoregions, a numerical artifact described in De Cáceres et al. (2010). That is why we divided plankton biodiversity into assemblages by using a cluster analysis, based on a Hellinger metric distance, jointly considering space and time (2‐month period) variability in the abundance of all plankton species/taxa. Therefore, an assemblage is here characterized by species/taxa exhibiting similar spatial and temporal patterns of abundance. This work has allowed us to complete the new biogeographic work of Beaugrand, Edwards, et al. (2019) made as part the European program ATLANTOS.

We identified 24 assemblages, each being characterized by their own degree of eurygraphy (Figure 2); some were truly eurygraphic (Figure 2c), while others were stenographic (Figure 2j); some were oceanic (Figure 2e), while others were more neritic or both; some were located south of the OPF, while others were detected north of the front. All together, they form a mosaic of taxonomic assemblages with overlapping spatial distribution in many different locations, a likely consequence of their environmental signatures. The examination of the spatial distribution of the 24 assemblages confirmed the existence of three main biomes in the North Atlantic (Figure 4): the Polar, the Westerlies, and the Coastal Boundary Zone Biomes (Longhurst, 1998). Each biome was identified by specific taxonomic assemblages having a specific environmental signature. North of the OPF, the Polar biome was characterized by Assemblages 10 and 6, with *Ceratium articum*, *Calanus glacialis*, *C. hyperboreus, and Heterorhabdus norvegicus* (Figure 4n,r). South of the OPF, the Westerlies biome was characterized by many assemblages such as Assemblages 24, 14, 20, and 8, with *Ceratium teres*, *Candacia ethiopica*, *Euchaeta marina,* and *Rhincalanus nasutus* (Figure 4h,k,m,o). Assemblages 13, 17, and 2 (Figure 4j,l,u) were more characteristic of the Coastal Boundary Zone biome, with *Odontella regia*, *O. mobiliensis*, *Paralia sulcata*, *Isias clavipes,* and *Labidocera wollastoni*.

Although many assemblages were found in a specific biome (sensu Longhurst, 1998), none were characteristic of a specific ecological unit sensu Beaugrand, Edwards, et al. (2019). In contrast to the terrestrial realm, lack of strong physical barriers associated with changing hydroclimatic conditions may partly explain this observation (Van der Spoel, 1994). Spatial coenoclines (i.e., gradients of biocoenoses or communities) we observed in our study (Figures 2, 3, 4) can be compared with annual succession observed in ecosystems during the year (Romagnan et al., 2015). These biological gradients in space and time are a perfect illustration of the ecological principle of impermanence resulting from the constant biological adjustments that originate from niche‐environment interaction (Beaugrand, Conversi, et al., 2019). Sharp or gradual environmental gradients interact with the niche of each species within a multidimensional space to generate a variety of biogeographic patterns.

We do not think that the overlapping spatial distribution of some assemblages with the partition proposed by Beaugrand, Edwards, et al. (2019) is due to differences in spatial resolution. Partitioning the pelagic ocean is difficult because of the absence of geographical barriers and because any partition changes over time from daily to decadal scales (Reygondeau et al., 2013). In addition, because of the principle of competitive exclusion of Gause (1934), we should expect all spatial ranges and phenologies to be unique (Caracciolo et al., 2021). The necessary corollary of this principle is that any synthetic partition should not be expected to work for all species or species assemblages. Temporal dynamics and plankton dispersal or expatriation are also mechanisms adding further complexity.

We found no obvious relationships between the spatial distribution of the species assemblages, seasonal patterns, and taxonomic composition in most cases (Figure 2 vs. Figure S5). We also found no obvious relationships between assemblages and taxonomic resolution (Figure S6). However, assemblages occurring in the same ecological units frequently have the same environmental signature, as Assemblages 13 and 17 (Figures 4j,l and 5j,l). We therefore suggest that the environmental signature of each assemblage drives their spatial and temporal patterns. Assemblages with a large environmental signature occurred in almost all the ecological units and thus had a large spatial distribution. This was the case for Assemblages 12 and 21 (Figures 4c,g and 5c,g) which had large environmental signature and occurred in the thirteen ecological units. Therefore, assemblages composed of euryoecious species were eurygraphic.

The environmental signature of a group was characterized by means of an environmental chromatogram. This procedure has enabled a rapid display of the 9‐dimensional environmental signature of the 24 assemblages into a two‐dimensional space (Figures 5 and S10). The environmental chromatogram separated plankton assemblages according to their degree of environmental tolerance (i.e., degree of euryoecy) and optima (e.g., degree of thermophily). We suggest that the method could also characterize the (multidimensional) ecological niche (sensu Hutchinson, 1957) of a species where both the amplitude and the optimum of an environmental factor would be represented as a function of the number of ecological dimensions, with the abundance (instead of a percentage of aggregation) as a third dimension (i.e., the color of the contour plot). The environmental chromatogram is a new method that enables one to display all multidimensional niches composing an assemblage (there are as many niches as species). Such chromatograms allow a rapid display of the environmental signature of an assemblage. Furthermore, the visual comparison of the chromatograms can immediately reveal the degree of overlapping of two environmental signatures and explain why the spatial distribution of two assemblages overlaps. For example, Assemblage 8 (Figure 2o) has a spatial distribution that is included inside Assemblage 5 (Figure 2s), a result that is expected from the environmental chromatogram of the two assemblages (Figure 5o,s). To our knowledge, there is no graphical method enabling a clear representation of a multidimensional ecological niche into more than two dimensions. A radar plot can be used but results are less easy to interpret, especially when a large number of species are considered (Reygondeau et al., 2012). Multivariate analyses have been sometimes used (Helaouët & Beaugrand, 2007), but because each component is a linear combination of different ecological factors, the resulting multidimensional niche is more difficult to interpret.

It has been shown that cold‐water species (e.g., *Calanus finmarchicus*, Assemblage 12) have a larger niche breadth than their warmer‐water counterparts (e.g., *Calanus helgolandicus*, Assemblage 19) (Sunday et al., 2011). Indeed, in our 24 assemblages, we noticed that few were specific of northern ecological units (e.g., Assemblages 10 and 6 in Figure 4n,r). Among them, many had a large spatial range covering sometimes the whole North Atlantic Ocean and its adjacent seas. We suggest that the spatial range of these assemblages illustrates well Rapoport's effect, which postulates that high‐latitude species have a larger geographical range than mid‐latitude species (Stevens, 1989).

## 5 CONCLUSIONS

Spatial patterns in plankton biodiversity are the result of environmental factors acting at both global and local scales (Beaugrand et al., 2000). We have decomposed CPR‐sampled plankton biodiversity into 24 taxonomic assemblages using both phytoplankton and zooplankton. These assemblages are interesting because they characterize specific hydroclimatic conditions and so they can be used as biological indicators, either of a substrate‐biotope (water mass) component or of a stable‐biotope (key area) component (Beaugrand et al., 2002). Their associated specific environmental signatures have allowed us to better characterize the ecosystems of the North Atlantic Ocean and its adjacent seas and to improve our understanding of the arrangement of plankton biodiversity.

Although some assemblages were characteristic of the three biomes observed in our studied areas, none of them were observed specifically in the ecological units identified by Beaugrand, Edwards, et al. (2019). This important result can in part be explained by the regional complexity of the northern part of the North Atlantic Ocean, which exhibits a pronounced hydrodynamic variability with interwoven substrate and stable‐biotope components. An alternative hypothesis is that it could be a universal feature. All species having a unique ecological niche after the principle of competitive exclusion of Gause (1934), the corollary is that they are expected to exhibit distinct spatial distributions. We have clearly shown that each taxonomic assemblage had a unique environmental signature and that therefore Gause's corollary can be extended at the community level. In the same way that coenoclines are observed during annual succession and that community reorganization takes place all the time from year‐to‐year to multidecadal scales, coenoclines may occur everywhere in the ocean. Pelagic ecosystems are therefore likely to be more complex than previously envisioned.

## CONFLICT OF INTEREST

The authors declare no conflicts of interest.

## AUTHOR CONTRIBUTION

**Loïck Kléparski:** Conceptualization (equal); Formal analysis (equal); Investigation (equal); Methodology (equal); Writing‐original draft (equal); Writing‐review & editing (equal). **Grégory Beaugrand:** Conceptualization (equal); Formal analysis (equal); Funding acquisition (equal); Investigation (equal); Methodology (equal); Supervision (equal); Writing‐original draft (equal); Writing‐review & editing (equal). **Martin Edwards:** Conceptualization (equal); Formal analysis (equal); Funding acquisition (equal); Investigation (equal); Methodology (equal); Supervision (equal); Writing‐original draft (equal); Writing‐review & editing (equal).

## Supporting information

Supplementary Material

## Data Availability

Data used in the production of this manuscript are already freely available (see Appendix 1).

## APPENDIX 1

All Continuous Plankton Recorder data is freely available on request by contacting the Marine Biological Association (MBA), United Kingdom. Data requests (Dan Lear: dble@mba.ac.uk).

Sea Surface Temperature (SST) originated from Centennial *in situ* Observation Based Estimates (COBE) SST2 dataset (https://www.esrl.noaa.gov/psd/) (Hirahara et al., 2014). Units were in degree Celsius.

Bathymetry data originated from the BIO‐ORACLE V2.0 dataset (Assis et al., 2017; Tyberghein et al., 2012). (http://www.bio‐oracle.org/). Bathymetry was expressed in meters.

Monthly mean Downward Solar Radiation Flux at surface (DSRF) originated from the National Centers for Environmental Prediction (NCEP), National Center for Atmospheric Research (NCAR), provided by the NOAA/OAR/ESRL PSD, Boulder Colorado, USA (https://www.esrl.noaa.gov/psd/data/gridded/data.ncep.reanalysis.html). We used this variable as a proxy of PAR (Photosynthetically Active Radiation) because it covered the northern part of our studied zone. Unit was in W/m^2^.

Nutrients data, originated from the World Ocean Database (WOD) (https://www.nodc.noaa.gov/OC5/WOD/pr_wod.html). We used three variables: (a) nitrate, (b) phosphate and (c) silicate concentration. Data were expressed in µmol/kg.

Distance to nearest coastline was provided by the University Corporation for Atmospheric Research (UCAR), community programs, Boulder Colorado, USA (http://oos.soest.hawaii.edu/thredds/ncss/dist2coast_1deg_ocean/dataset.html). Units were in kilometres.

Density Mix Layer Depth (DMLD) originated from Monthly Isopycnal and Mixed‐layer Ocean Climatology (MIMOC) provided by NOAA (Johnson et al., 2012). Units were in dbar.

Sea Surface Salinity (SSS) originated from NCPE Global Ocean Data Assimilation System (GODAS) dataset, provided by NOAA ESRL, Physical sciences division, Boulder Colorado, USA. Units were in kg/kg. (https://www.esrl.noaa.gov/psd/).
