# Supplementary material for: Plankton biogeography in the North Atlantic Ocean and its adjacent seas: Species assemblages and environmental signatures
Source: Ecol Evol. 2021 Mar 24;11(10):5135–49. doi: 10.1002/ece3.7406 (PMC8131763; doi:10.1002/ece3.7406)
Supplement: Supplementary file 1 — Supplementary Material [file ECE3-11-5135-s001.docx]

**Supplementary Information**

**Plankton biogeography in the North Atlantic Ocean and its adjacent seas: species assemblages and environmental signatures**

Loïck Kléparski^1,3^, Grégory Beaugrand^2,3^, and Martin Edwards^3,4^

*^1^Univ. Littoral Côte d’Opale, CNRS, Univ. Lille, UMR 8187 - LOG - Laboratoire d’Océanologie et de Géosciences, F-62930 Wimereux, France.*

*^2^Univ. Lille, CNRS, Univ. Littoral Côte d’Opale, UMR 8187 - LOG - Laboratoire d’Océanologie et de Géosciences, F-59000 Lille, France.*

*^3^Continuous Plankton Recorder (CPR) survey, the Marine Biological Association, Citadel Hill, Plymouth PL1 2PB, UK.*

*^4^Marine Institute, Plymouth University, Drake Circus, Plymouth, PL4 8AA, UK.*

**Supplementary tables and figures**

**SUPPLEMENTARY TABLES**

**Table S1.** List of species/taxa composing each assemblage.

| Assemblage | Species/taxa | |
| --- | --- | --- |
| 1 | *Ceratium platycorne* | Dinoflagellates |
|  | *Ceratium vultur* | Dinoflagellates |
|  | *Phalacroma* spp. | Dinoflagellates |
|  | *Candacia longimana* | Copepoda |
|  | *Diacria* spp. | Gastropoda |
|  | *Lepas cypris* | Cirripedia |
|  | *Paraeuchaeta gracilis* | Copepoda |
|  | *Ctenocalanus vanus* | Copepoda |
| 2 | *Bellerochea malleus* | Diatom |
|  | *Guinardia flaccida* | Diatom |
|  | *Labidocera wollastoni* | Copepoda |
|  | *Isias clavipes* | Copepoda |
| 3 | *Pyrophacus* spp. | Dinoflagellates |
|  | *Biddulphia alternans* | Diatom |
|  | *Pterosperma* spp. (Total) | Prymnesiophyceae |
|  | *Cystodinium* spp. | Dinoflagellates |
|  | *Corythodinium* spp. | Dinoflagellates |
|  | *Gyrodinium* spp. | Dinoflagellates |
|  | *Prorocentrum micans* | Dinoflagellates |
|  | *Branchiostoma lanceolatum* | Cephalochordata |
|  | *Euterpina acutifrons* | Copepoda |
|  | *Zoothamnium pelagicum* | Ciliophora |
|  | Spindelei | Trematoda |
|  | Clytemnestridae | Copepoda |
| 4 | *Detonula pumila* | Diatom |
|  | *Calanoides carinatus* | Copepoda |
|  | *Centropages chierchiae* eyecount | Copepoda |
|  | *Centropages chierchiae* traverse | Copepoda |
| 5 | *Candacia armata* | Copepoda |
|  | *Echinoderm* post larvae | Echinodermata |
|  | *Subeucalanus crassus* | Copepoda |
|  | *Paraeuchaeta hebes* | Copepoda |
|  | *Corycaeus* spp. | Copepoda |
| 6 | *Rhizosolenia acuminata* | Diatom |
|  | *Proboscia curvirostris* | Diatom |
|  | *Aetideus armatus* | Copepoda |
|  | *Heterorhabdus norvegicus* | Copepoda |
|  | *Scolecithricella* spp. | Copepoda |
| 7 | *Ceratium buceros* | Dinoflagellates |
|  | *Ceratium lunula* | Dinoflagellates |
| 8 | *Ceratium candelabrum* | Dinoflagellates |
|  | *Ceratium carriense* | Dinoflagellates |
|  | *Ceratium declinatum* | Dinoflagellates |
|  | *Ceratium extensum* | Dinoflagellates |
|  | *Ceratium massiliense* | Dinoflagellates |
|  | *Ceratium pentagonum* | Dinoflagellates |
|  | *Ceratium teres* | Dinoflagellates |
|  | *Ceratium trichoceros* | Dinoflagellates |
| 9 | *Pyrocystis* spp. | Dinoflagellates |
|  | *Guinardia cylindrus* | Diatom |
|  | *Microcalanus* spp. | Copepoda |
| 10 | *Ceratium longipes* | Dinoflagellates |
|  | *Ceratium arcticum* | Dinoflagellates |
|  | *Fragilaria* spp. | Diatom |
|  | *Calanus glacialis* | Copepoda |
|  | *Calanus hyperboreus* | Copepoda |
|  | *Metridia longa* | Copepoda |
|  | *Parafavella gigantea* | Ciliophora |
|  | *Ptychocylis* spp. | Ciliophora |
| 11 | *Neocalyptrella robusta* | Diatom |
|  | Pycnogonida | Arthropoda |
| 12 | *Thalassiosira* spp. | Diatom |
|  | *Rhizosolenia styliformis* | Diatom |
|  | *Chaetoceros(Hyalochaete)* spp*.* | Diatom |
|  | *Chaetoceros(Phaeoceros)* spp*.* | Diatom |
|  | *Thalassiothrix longissima* | Diatom |
|  | *Thalassionema nitzschioides* | Diatom |
|  | *Ceratium fusus* | Dinoflagellates |
|  | Coccolithaceae (Total) | Prymnesiophyceae |
|  | Silicoflagellates | Prymnesiophyceae |
|  | *Pseudo-nitzschia delicatissima complex* | Diatom |
|  | *Pseudo-nitzschia seriata complex* | Diatom |
|  | *Calanus finmarchicus* | Copepoda |
|  | Hyperiidea (Total) | Malacostraca |
|  | Euphausiacea Adult | Malacostraca |
|  | Euphausiacea Juvenile | Malacostraca |
|  | *Paraeuchaeta norvegica* | Copepoda |
|  | *Para-Pseudocalanus* spp. | Copepoda |
|  | *Oithona* spp. | Copepoda |
|  | Calanus Total Traverse | Copepoda |
|  | Foraminifera (Total) | Protozoa |
|  | Appendicularia | Urochordata |
|  | Tintinnida Total | Ciliophora |
|  | Thecosomata (North Atlantic) | Gastropoda |
| 13 | *Actinoptychus* spp. | Diatom |
|  | *Odontella aurita* | Diatom |
|  | *Odontella granulata* | Diatom |
|  | *Odontella regia* | Diatom |
|  | *Odontella mobiliensis* | Diatom |
|  | *Rhizosolenia pungens* | Diatom |
|  | *Asterionellopsis glacialis* | Diatom |
|  | *Bleakeleya notata* | Diatom |
|  | *Bacillaria paxillifera* | Diatom |
|  | Ammodytidae | Actinopterygii |
|  | *Hemicyclops aberdonensis* | Copepoda |
| 14 | *Phaeocystis pouchetii* | Prymnesiophycea |
|  | *Ceratium compressum* | Dinoflagellates |
|  | *Nitzschia bicapitata* | Diatom |
|  | *Rhincalanus nasutus* | Copepoda |
|  | *Eucalanus hyalinus* | Copepoda |
|  | *Pneumodermopsis ciliata* | Gastropoda |
|  | Thaliacea | Urochordata |
|  | Salpidae (Total) | Urochordata |
|  | Doliolidae | Urochordata |
|  | Cnidaria tissue | Cnidaria |
| 15 | *Neocalanus gracilis* | Copepoda |
|  | *Nannocalanus minor* | Copepoda |
|  | *Euchirella rostrata* | Copepoda |
|  | *Euchaeta acuta* | Copepoda |
|  | *Pleuromamma abdominalis* | Copepoda |
|  | *Pleuromamma borealis* | Copepoda |
|  | *Pleuromamma gracilis* | Copepoda |
|  | *Centropages bradyi* | Copepoda |
|  | *Heterorhabdus papilliger* | Copepoda |
|  | *Pleuromamma piseki* | Copepoda |
|  | *Pleuromamma xiphias* | Copepoda |
|  | *Sapphirina* spp. | Copepoda |
|  | *Undeuchaeta plumosa* | Copepoda |
|  | Sergestidae | Malacostraca |
|  | *Mesocalanus tenuicornis* | Copepoda |
|  | *Calocalanus* spp. | Copepoda |
|  | *Lucicutia* spp. | Copepoda |
|  | *Mecynocera clausi* | Copepoda |
|  | *Oncaea* spp. | Copepoda |
| 16 | *Asteromphalus* spp. | Diatom |
|  | *Cerataulina pelagica* | Diatom |
|  | *Climacodium frauenfeldianum* | Diatom |
|  | *Detonula confervacea* | Diatom |
|  | *Leptocylindrus danicus* | Diatom |
|  | *Rhaphoneis amphiceros* | Diatom |
|  | *Planktoniella* sol | Diatom |
|  | *Stephanopyxis* spp. | Diatom |
|  | *Surirella* spp. | Diatom |
|  | *Pachysphaera* spp. | Chlorodendrophyceae |
|  | *Amphisolenia* spp. | Dinoflagellates |
|  | *Ceratium arietinum* | Dinoflagellates |
|  | *Ceratium bucephalum* | Dinoflagellates |
|  | *Ceratium lamellicorne* | Dinoflagellates |
|  | *Ceratium pulchellum* | Dinoflagellates |
|  | *Pronoctiluca pelagica* | Dinoflagellates |
|  | *Ptychodiscus noctiluca* | Dinoflagellates |
|  | *Ornithocercus* spp. | Dinoflagellates |
|  | *Glenodinium* spp. | Dinoflagellates |
|  | *Gymnodinium* spp. | Dinoflagellates |
|  | *Katodinium* spp. | Dinoflagellates |
|  | *Hexasterias problematica* | Prasinophyceae |
|  | *Diploneis* spp. | Diatom |
|  | *Nitzschia sigma rigida* | Diatom |
|  | *Actiniscus pentasterias* | Dinoflagellates |
|  | *Nitzschia longissima* | Diatom |
|  | *Neodenticula seminae* | Diatom |
|  | *Dinophysis acuminata* | Dinoflagellates |
|  | *Dinophysis acuta* | Dinoflagellates |
|  | *Dinophysis caudata* | Dinoflagellates |
|  | *Dinophysis norvegica* | Dinoflagellates |
|  | *Dinophysis tripos* | Dinoflagellates |
|  | *Bacterosira bathyomphala* | Diatom |
|  | *Prorocentrum rostratum* | Dinoflagellates |
|  | *Prorocentrum dentatum* | Dinoflagellates |
|  | *Phalacroma rotundatum* | Dinoflagellates |
|  | *Podosira stelligera* | Diatom |
|  | *Pseudosolenia calcar-avis* | Diatom |
|  | *Helicotheca tamesis* | Diatom |
|  | *Mediopyxis helysia* | Khakista |
|  | *Membraneis* spp. | Diatom |
|  | *Scaphocalanus echinatus* | Copepoda |
|  | *Euchaeta media* | Copepoda |
|  | *Paracandacia bispinosa* | Copepoda |
|  | *Rhincalanus cornutus* | Copepoda |
|  | *Undeuchaeta major* | Copepoda |
|  | *Labidocera aestiva* | Copepoda |
|  | *Paedoclione doliiformis* | Gastropoda |
|  | Isopoda (Total) | Malacostraca |
|  | *Cavolinia* spp. | Gastropoda |
|  | *Clio* spp. | Gastropoda |
|  | *Pneumodermopsis paucidens* | Gastropoda |
|  | *Oxygyrus* spp. | Gastropoda |
|  | *Peraclis* spp. | Gastropoda |
|  | Stomatopoda | Malacostraca |
|  | *Alteutha* spp. | Arthropoda |
|  | *Lucifer* spp. | Malacostraca |
|  | Pipefish | Fish |
|  | *Siphonostomatoida* | Copepoda |
|  | *Parathalestris croni* | Copepoda |
|  | *Penilia avirostris* | Cladocera |
|  | *Favella serrata* | Ciliophora |
|  | *Acartia danae* | Copepoda |
|  | *Acrocalanus* spp. | Copepoda |
|  | *Lubbockia* spp. | Copepoda |
|  | *Parapontella brevicornis* | Copepoda |
|  | *Tortanus discaudatus* | Copepoda |
|  | *Acartia longiremis* | Copepoda |
|  | Euphausiacea eggs | Malacostraca |
|  | Rotifer eggs | Rotifera |
| 17 | *Paralia sulcata* | Diatom |
|  | *Odontella sinensis* | Diatom |
|  | *Gyrosigma* spp. | Diatom |
|  | *Noctiluca scintillans* | Dinoflagellates |
|  | *Coscinodiscus wailesii* | Diatom |
|  | Gammaridea | Malacostraca |
|  | Cumacea | Malacostraca |
|  | Mysidacea | Malacostraca |
|  | Caprelloidea | Malacostraca |
| 18 | *Ceratium belone* | Dinoflagellates |
|  | *Ceratium gibberum* | Dinoflagellates |
|  | *Ceratium inflatum* | Dinoflagellates |
|  | *Ceratium karstenii* | Dinoflagellates |
|  | *Histioneis* spp. | Dinoflagellates |
|  | *Goniodoma polyedricum* | Dinoflagellates |
| 19 | *Ditylum brightwellii* | Diatom |
|  | *Eucampia zodiacus* | Diatom |
|  | *Rhizosolenia setigera* | Diatom |
|  | *Guinardia delicatula* | Diatom |
|  | *Dactyliosolen fragilissimus* | Diatom |
|  | *Guinardia striata* | Diatom |
|  | *Lauderia annulata* | Diatom |
|  | *Calanus helgolandicus* | Copepoda |
|  | Decapoda larvae (Total) | Malacostraca |
|  | Fish eggs (Total) | Fish |
|  | *Anomalocera patersoni* | Copepoda |
|  | *Temora longicornis* | Copepoda |
|  | *Centropages hamatus* | Copepoda |
|  | Cyphonautes | Bryozoa |
|  | Echinoderm larvae | Echinodermata |
|  | Cirripede larvae (Total) | Cirripedia |
|  | *Tintinnopsis* spp. | Ciliophora |
|  | Bivalvia *larvae* | Mollusca |
|  | *Pseudocalanus* spp. Adult Atlantic | Copepoda |
| 20 | *Hemiaulus* spp. | Diatom |
|  | *Ceratium setaceum* | Dinoflagellates |
|  | *Ceratocorys* spp. | Dinoflagellates |
|  | *Cladopyxis* spp. | Dinoflagellates |
|  | *Centropages violaceus* | Copepoda |
|  | *Copilia* spp. | Copepoda |
|  | *Atlanta* spp. | Heteropoda |
|  | *Temora stylifera* | Copepoda |
| 21 | *Skeletonema costatum* | Diatom |
|  | *Rhizosolenia hebetata semispina* | Diatom |
|  | *Coscinodiscus concinnus* | Diatom |
|  | *Navicula* spp. | Diatom |
|  | *Cylindrotheca closterium* | Diatom |
|  | *Proboscia inermis* | Diatom |
|  | *Ephemera planamembranacea* | Diatom |
|  | *Corethron hystrix* | Diatom |
|  | *Tomopteris* spp. | Annelida |
|  | *Clione limacina* | Gastropoda |
|  | Fish larvae | Fish |
|  | Ostracoda | Ostracoda |
|  | Cephalopoda larvae | Cephalopoda |
|  | *Halosphaera* spp. | Prymnesiophyceae |
|  | *Euphausiacea calyptopis* | Malacostraca |
|  | *Euphausiacea nauplii* | Malacostraca |
| 22 | *Ceratium furca* | Dinoflagellates |
|  | *Ceratium lineatum* | Dinoflagellates |
|  | *Ceratium tripos* | Dinoflagellates |
|  | *Ceratium macroceros* | Dinoflagellates |
|  | *Ceratium horridum* | Dinoflagellates |
|  | *Dinophysis* spp. Total | Dinoflagellates |
|  | *Protoperidinium* spp. | Dinoflagellates |
|  | *Prorocentrum* spp. Total | Dinoflagellates |
|  | *Scrippsiella* spp. | Dinoflagellates |
|  | *Proboscia alata* | Diatom |
|  | *Rhizosolenia imbricata* | Diatom |
|  | Chaetognatha eyecount | Chaetognatha |
|  | *Centropages typicus* | Copepoda |
|  | *Podon* spp. | Cladocera |
|  | *Evadne* spp. | Cladocera |
|  | Chaetognatha Traverse | Chaetognatha |
| 23 | *Dactyliosolen antarcticus* | Diatom |
|  | *Bacteriastrum* spp. | Diatom |
|  | *Rhizosolenia bergonii* | Diatom |
|  | *Ceratium azoricum* | Dinoflagellates |
|  | *Ceratium hexacanthum* | Dinoflagellates |
|  | *Ceratium minutum* | Dinoflagellates |
|  | *Gonyaulax* spp. | Dinoflagellates |
|  | *Oxytoxum* spp. | Dinoflagellates |
|  | *Podolampas* spp. | Dinoflagellates |
|  | *Leptocylindrus mediterraneus* | Diatom |
|  | *Prorocentrum* spp. ('Exuviaella' type) | Dinoflagellates |
|  | *Trichodesmium* spp. | Prymnesiophycea |
|  | *Proboscia indica* | Diatom |
|  | *Metridia lucens* | Copepoda |
|  | *Pleuromamma robusta* | Copepoda |
|  | *Clausocalanus* spp. | Copepoda |
|  | *Dictyocysta* spp. | Ciliophora |
|  | Harpacticoida Total Traverse | Copepoda |
|  | Metridia Total traverse | Copepoda |
|  | Radiolaria Total | Protozoa |
|  | *Acantharia* spp. | Protozoa |
|  | Radiolaria non-Acantharian | Protozoa |
|  | *Paracalanus* spp. | Copepoda |
|  | *Microsetella* spp. | Copepoda |
| 24 | *Miracia efferata* | Copepoda |
|  | *Urocorycaeus* spp. | Copepoda |
|  | *Candacia bipinnata* | Copepoda |
|  | *Candacia ethiopica* | Copepoda |
|  | *Candacia pachydactyla* | Copepoda |
|  | *Euchaeta marina* | Copepoda |
|  | *Scolecithrix danae* | Copepoda |
|  | *Undinula vulgaris* | Copepoda |
|  | *Paracandacia simplex* | Copepoda |
|  | Siphonophora | Cnidaria |
|  | *Lepas nauplii* | Cirripedia |
|  | *Corycaeus speciosus* | Copepoda |

**Table S2.** Spearman correlation coefficient (and its probability) between the environmental signature of each assemblage of phytoplankton and zooplankton. na: not applicable.

| Assemblage number | r | probability |
| --- | --- | --- |
| 1 | 0.43 | 0 |
| 2 | 0.70 | 0 |
| 3 | 0.66 | 0 |
| 4 | 0.38 | 0 |
| 5 | na | na |
| 6 | 0.58 | 0 |
| 7 | na | 0 |
| 8 | na | na |
| 9 | 0.17 | 0 |
| 10 | 0.73 | 0 |
| 11 | 0.29 | 0 |
| 12 | 0.71 | 0 |
| 13 | 0.55 | 0 |
| 14 | 0.70 | 0 |
| 15 | na | na |
| 16 | 0.87 | 0 |
| 17 | 0.85 | 0 |
| 18 | na | na |
| 19 | 0.79 | 0 |
| 20 | 0.68 | 0 |
| 21 | 0.76 | 0 |
| 22 | 0.90 | 0 |
| 23 | 0.85 | 0 |
| 24 | na | na |

**SUPPLEMENTARY FIGURES**

**Figure S1.** Spatial distribution of the ecological units identified by Beaugrand *et al.,* 2019 (1).


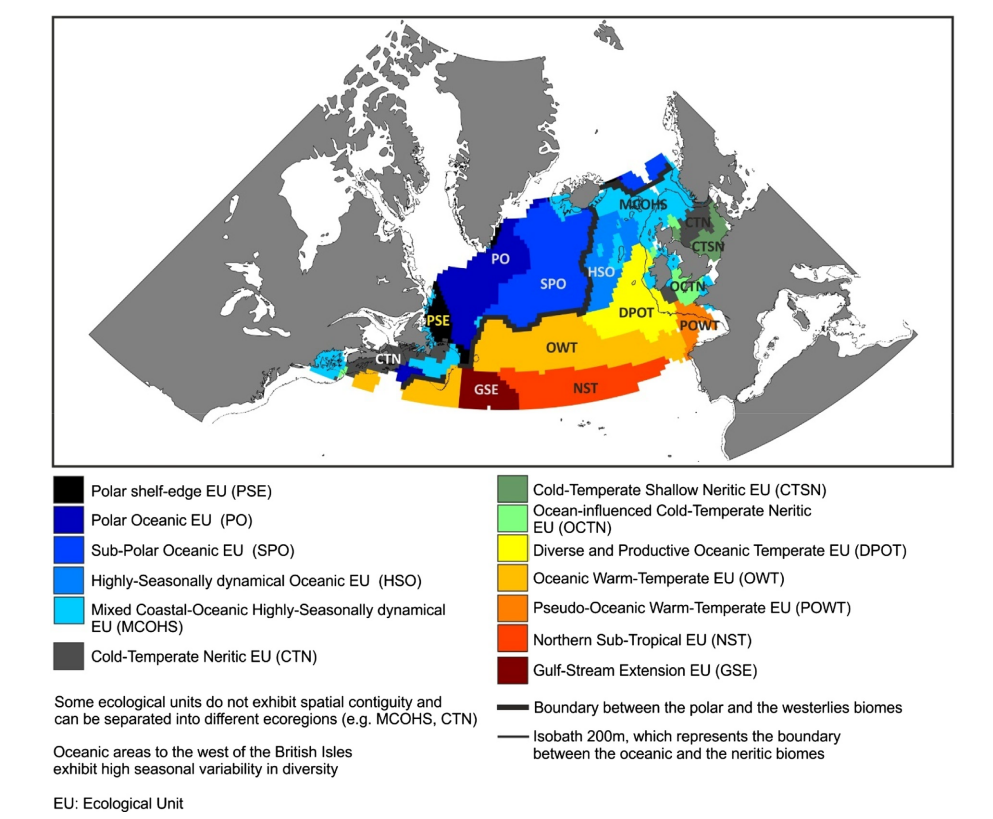


**Figure S2.** Spatial distribution of the ecoregions (panel a) and the ecological units (panel b) of Beaugrand *et al.,* (see also Figure S1). Adapted from Beaugrand *et al.,*2019 (1).

**
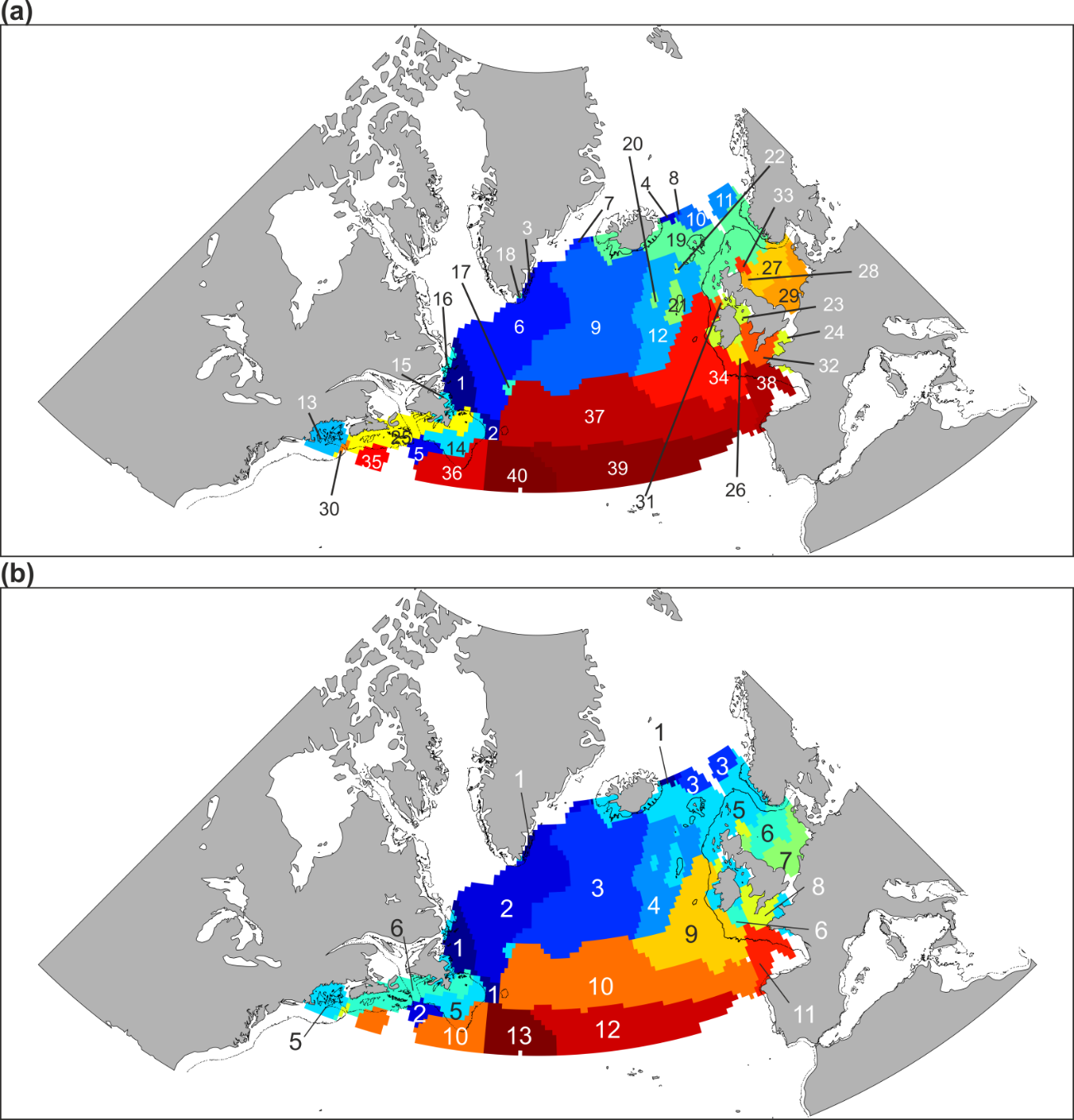
**

**Figure S3.** Sketch diagram that summarises all analyses performed in this study.


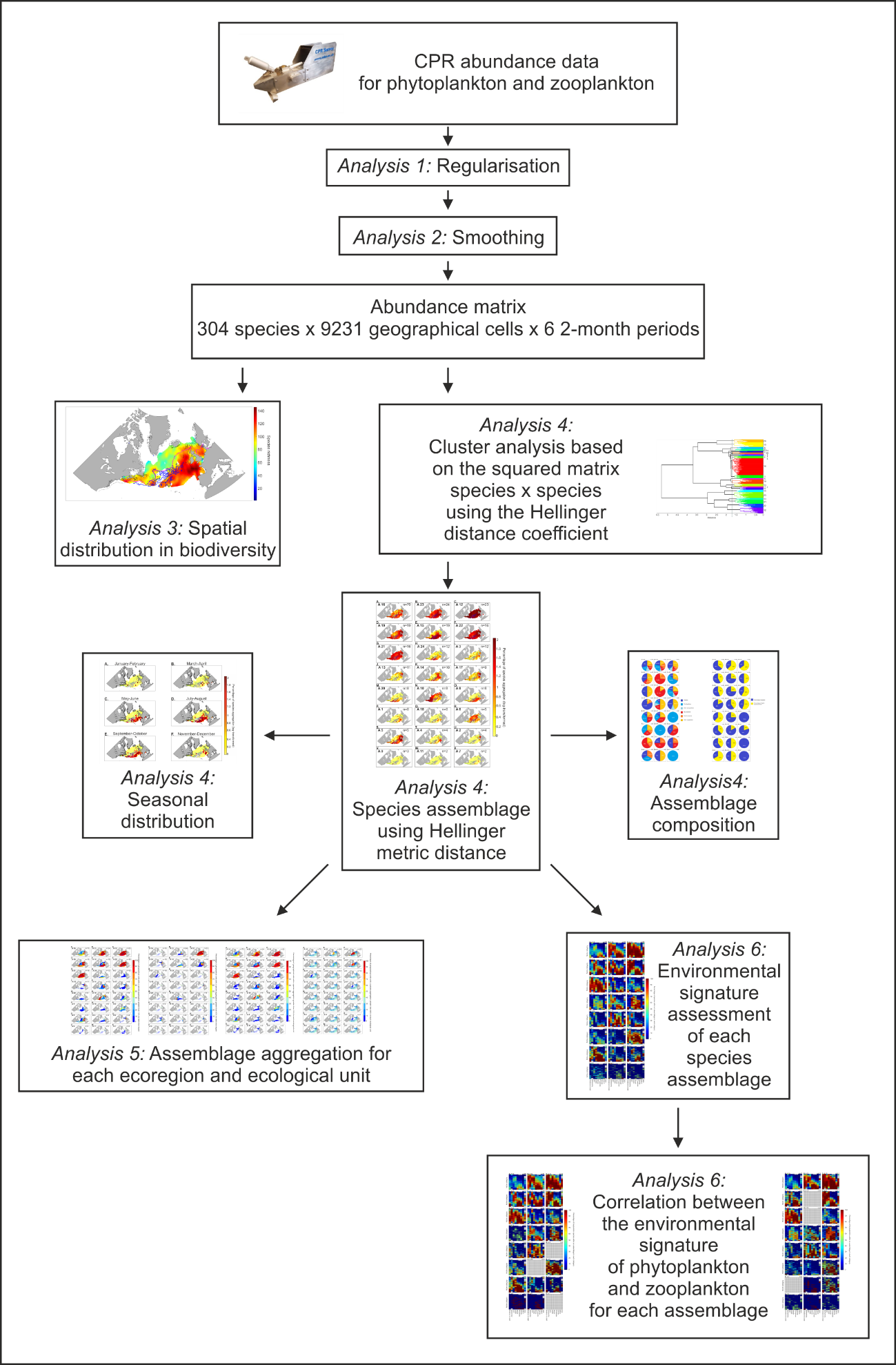


**Figure S4.** Dendrogram based on a cluster analysis performed on 304 species (or taxa) x 9231 geographical cells x 6 2-month period matrix using Ward’s algorithm applied on squared matrix of Hellinger distance. A total of 24 assemblages was found at the cut-off level of 1.7. Numbers on the right correspond to the assemblage number displayed in Figure 2. The grey dashed line denotes the position of the cut off level. Colours enable the distinction of a taxonomic assemblage.


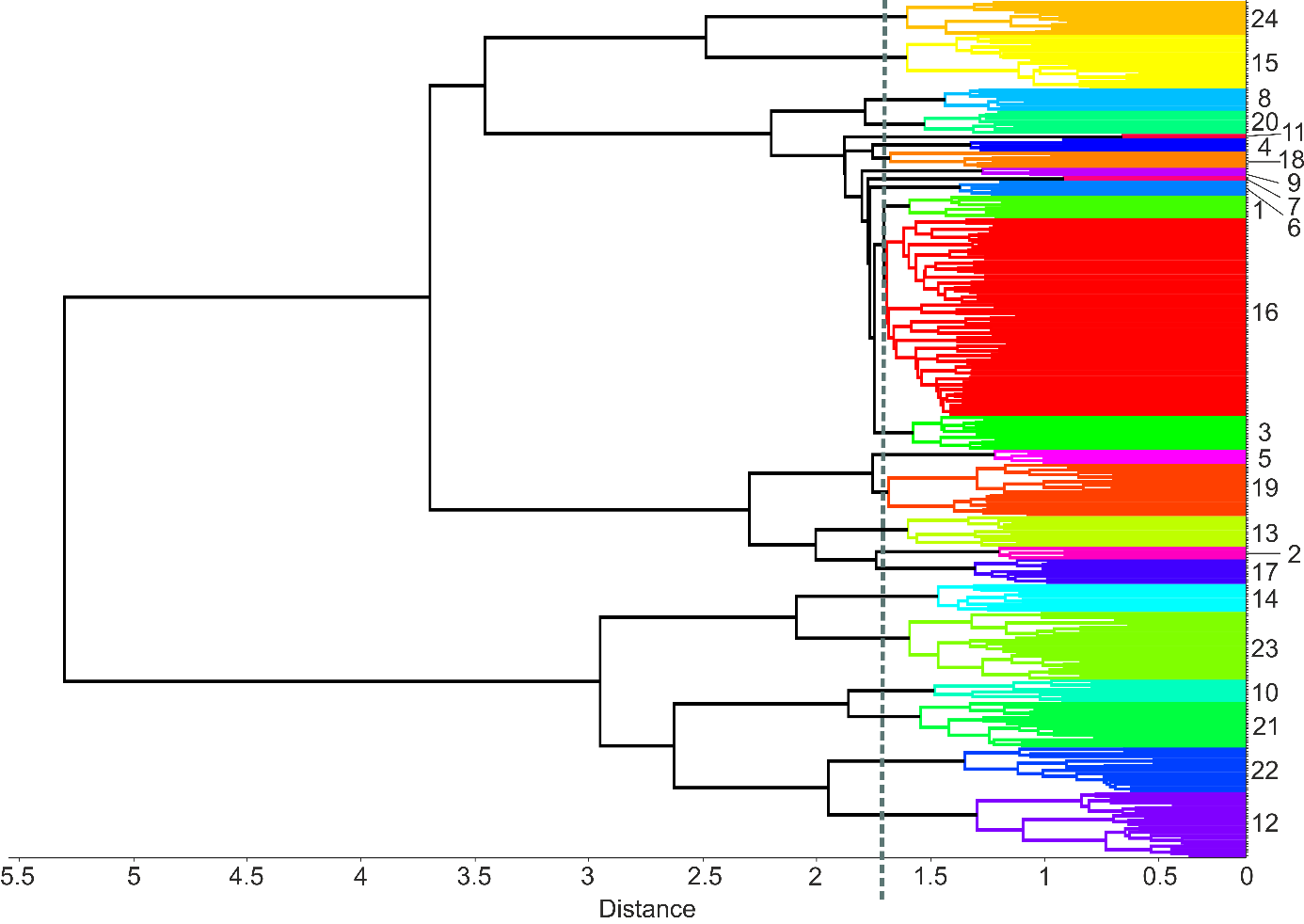


**Figure S5.** Taxonomic composition of the 24 assemblages in Figure 2 expressed as a percentage. Each colour corresponds to one of the taxonomic groups: diatoms, dinoflagellates, other phytoplankton, large copepods, small copepods and other zooplankton. Panels are classified from a to x by decreasing taxonomic richness. The number at the top left of each panel corresponds to the assemblage number (see Figure 2 and Figure S4) and the number at the top right (n) indicates the number of species (or taxa) of each assemblage.


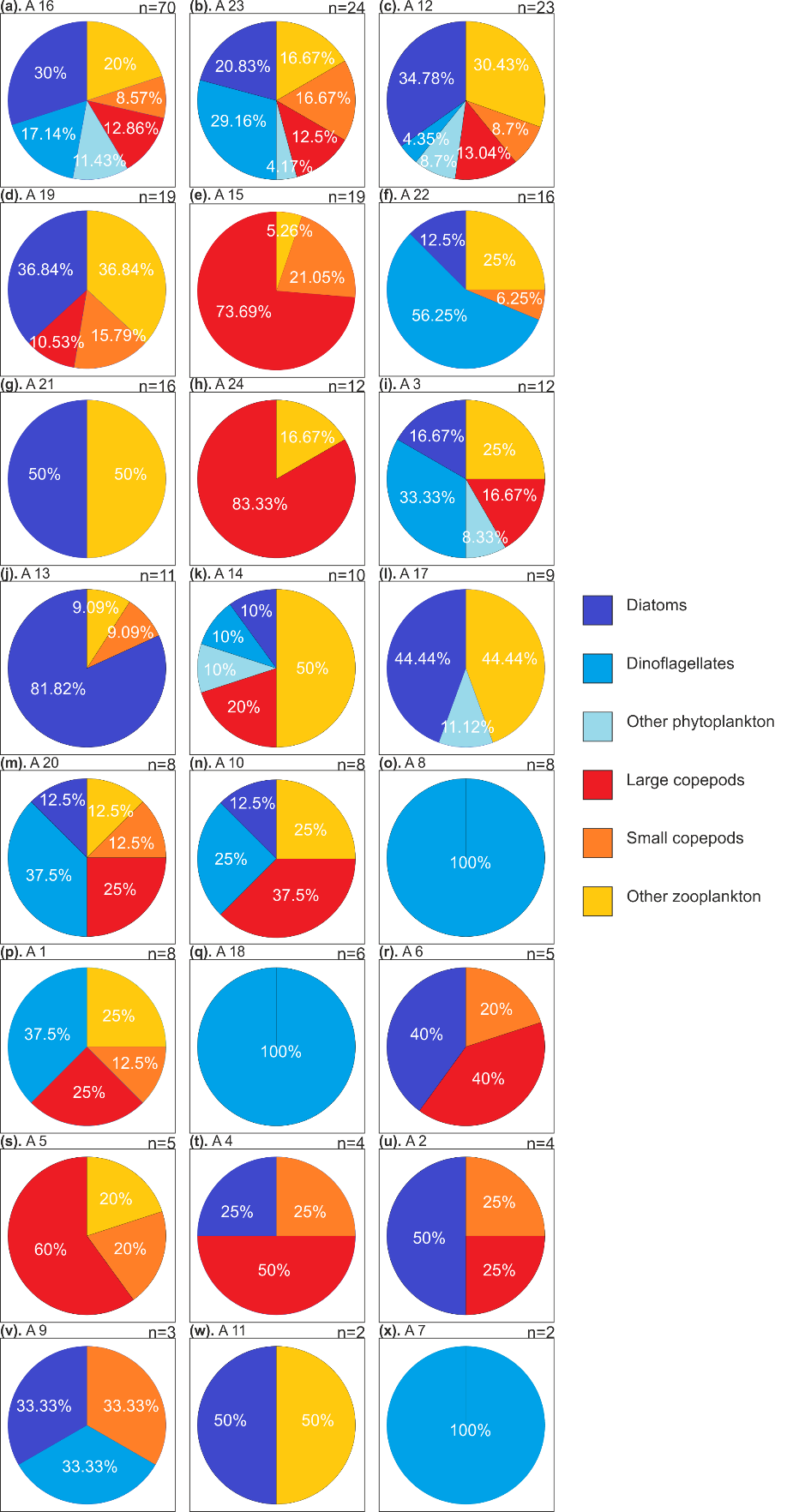


**Figure S6.** Assemblage composition considering only two taxonomic levels: species and coarser taxonomic resolution. Blue colour corresponds to the percentage of taxonomic units identified at the species level and yellow colour corresponds to the percentage of taxonomic units identified at a higher than the species level. As in Figure 2, panels (i.e. assemblages) are classified by decreasing taxonomic richness. The number at the top left of each panel corresponds to the assemblage number (see Figure 2 and Figure S4) and the number at the top right (n) indicates the taxonomic richness of each assemblage.

**
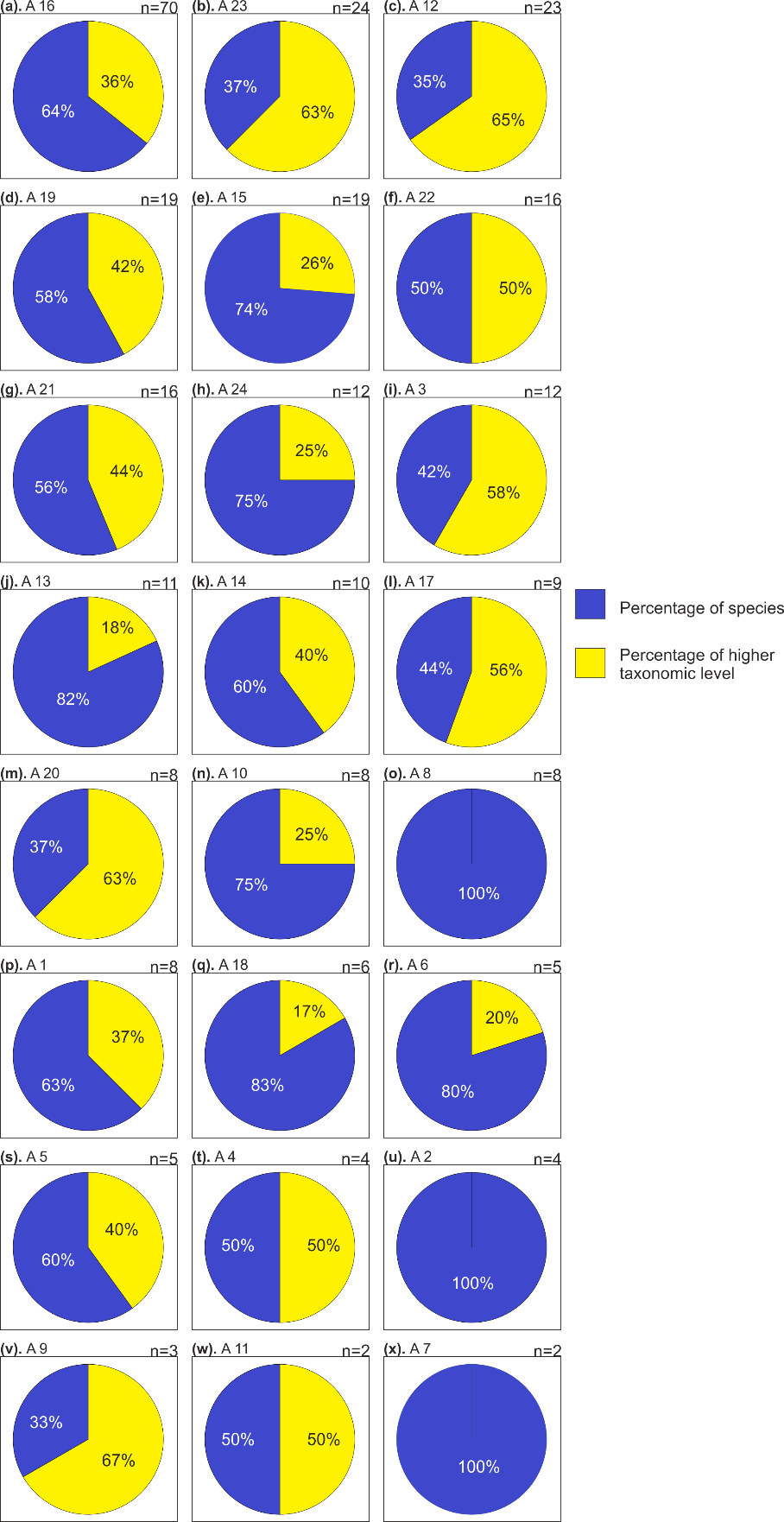
**

**Figure S7.** Percentage of taxonomic aggregation (i.e. number of species or taxa of the same assemblage) greater than 10% in each ecoregion as defined by Beaugrand *et al.,* (2019) (1). Here, the percentage of species/taxa aggregation is used to identify an assemblage characteristic of an ecoregion. On the top right of each panel, the number of species/taxa (n) in the assemblage is indicated. The assemblage number, corresponding to the numbers in Figure S4 and in Figure 2, is displayed in bold on the top left. The green line corresponds to isotherm 10°C. The blue lines correspond to current velocities from 0.5 to 2 m.s^-1^. The black lines denote isobath 200 m. Panels are sorted from a to x by decreasing taxonomic richness.


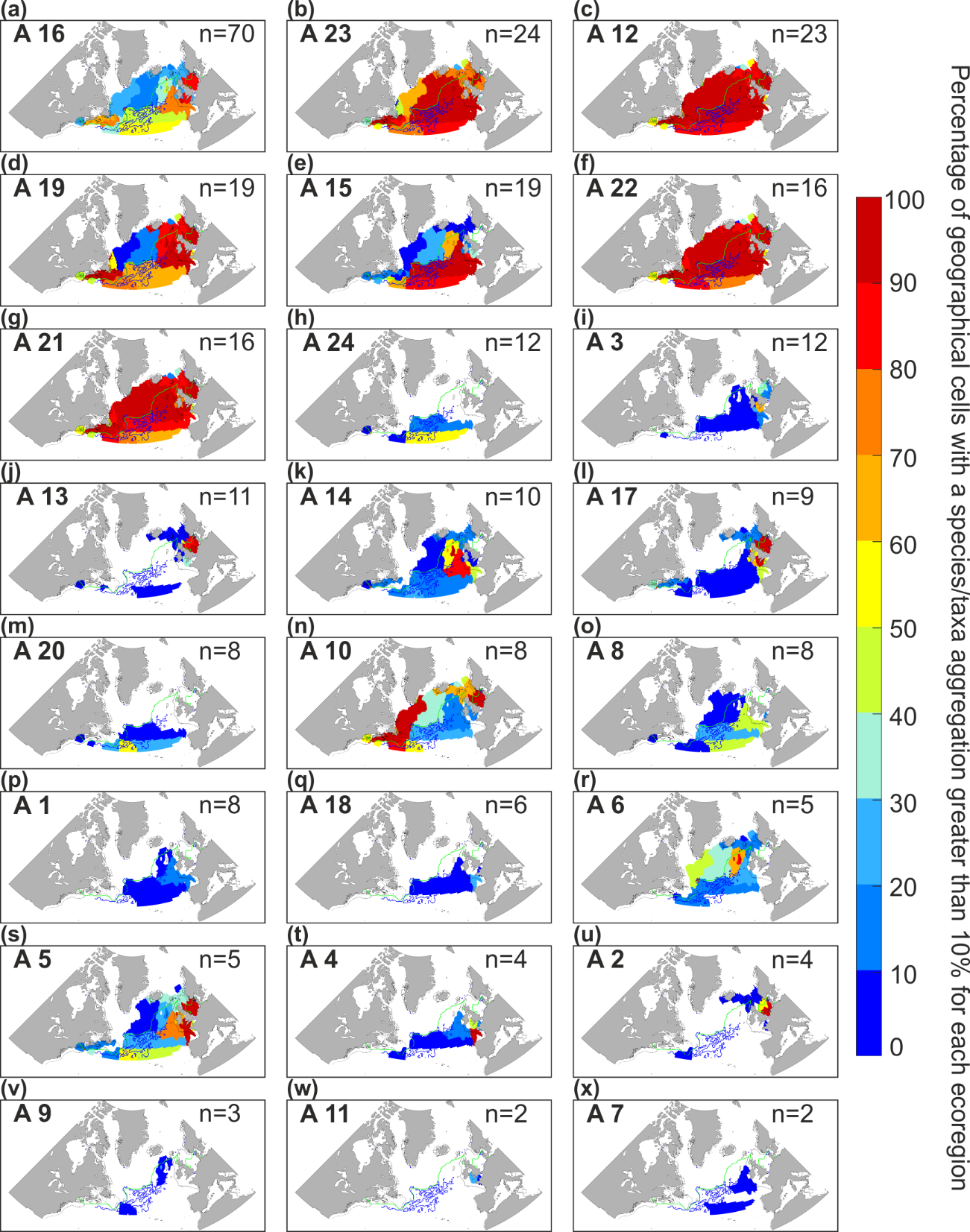


**Figure S8.** Percentage of taxonomic aggregation (i.e. number of species or taxa of the same assemblage) greater than 50% in each ecoregion as defined by Beaugrand *et al.,* (2019) (1). Here, the percentage of species/taxa aggregation is used to identify an assemblage characteristic of an ecoregion. On the top right of each panel, the number of species/taxa (n) in the assemblage is indicated. The assemblage number, corresponding to the numbers Figure S4 and in Figure 2, is displayed in bold on the top left. The green line corresponds to isotherm 10°C. The blue lines correspond to current velocities from 0.5 to 2 m.s^-1^. The black lines denote isobath 200 m. Panels are sorted from a to x by decreasing taxonomic richness.


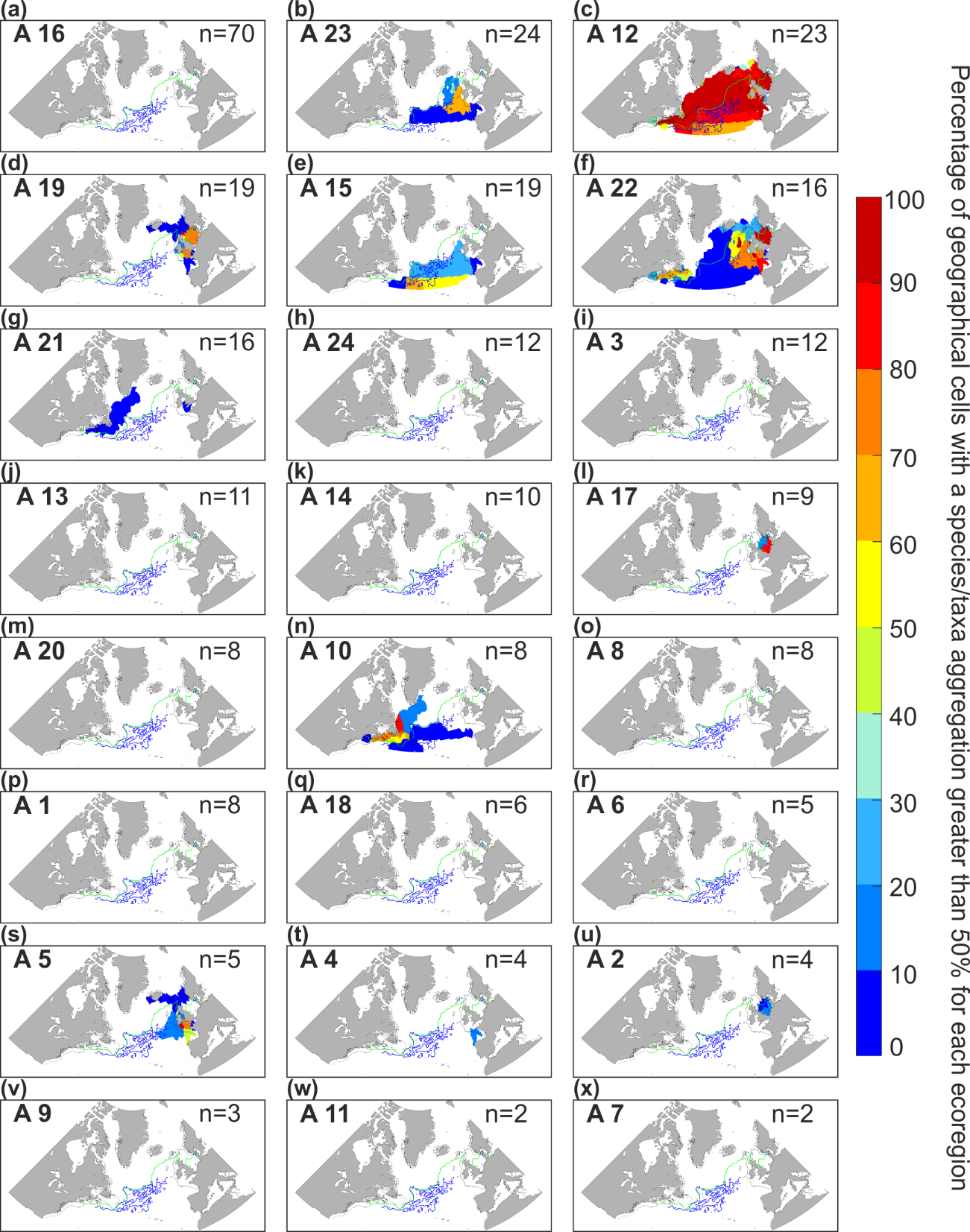


**Figure S9.** Percentage of taxonomic aggregation (i.e. number of species or taxa of the same assemblage) greater than 50% in each ecological unit as defined by Beaugrand *et al.,* (2019) (1). Here, the percentage of species/taxa aggregation is used to identify an assemblage characteristic of an ecological unit. On the top right of each panel, the number of species/taxa (n) in the assemblage is indicated. The assemblage number, corresponding to the numbers in Figure S4 and in Figure 2, is displayed in bold on the top left. The green line corresponds to isotherm 10°C. The blue lines correspond to current velocities from 0.5 to 2 m.s^-1^. The black lines denote isobath 200 m. Panels are sorted from a to x by decreasing taxonomic richness.


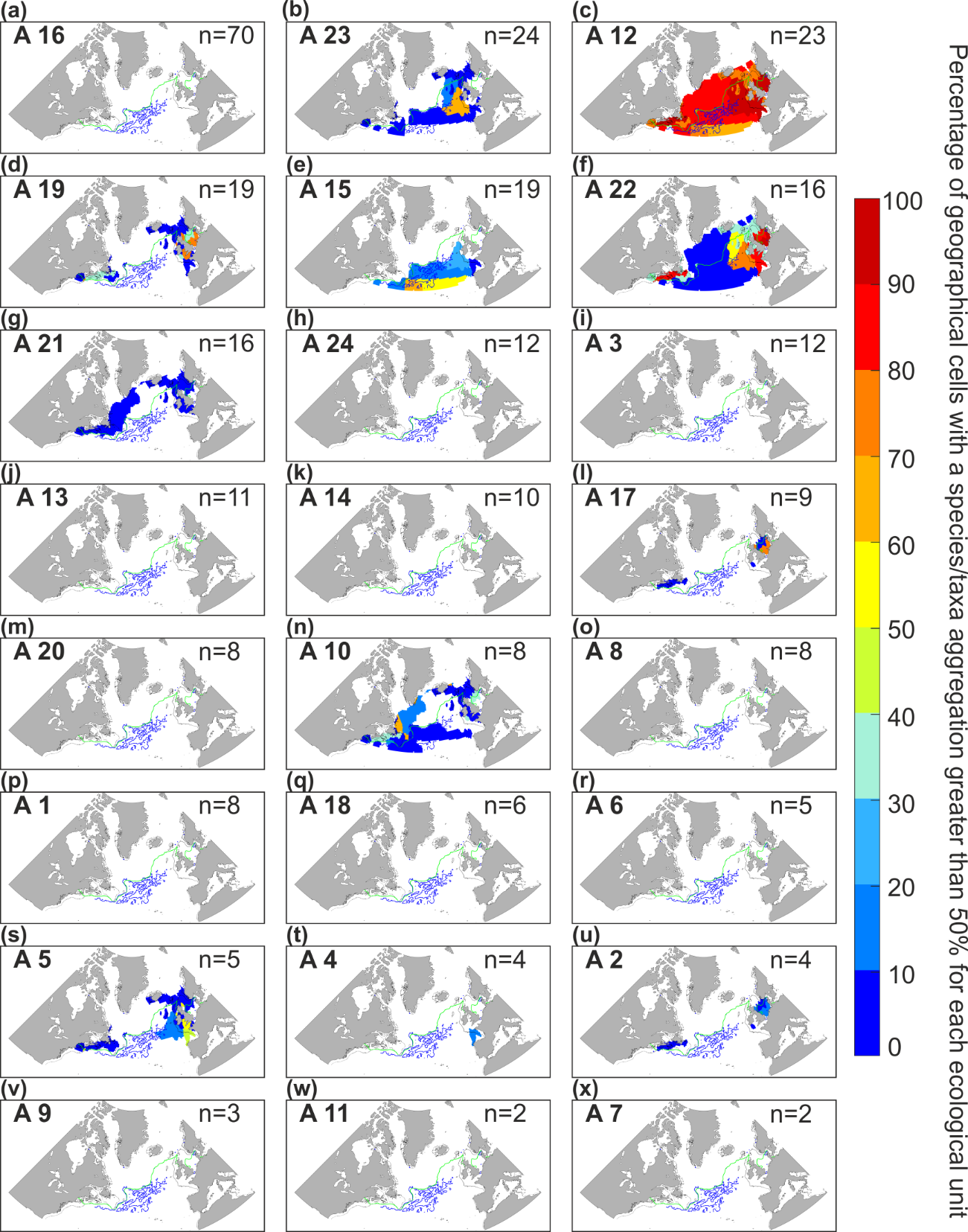


**Figure S10.** Environmental signature of phytoplankton species/taxa (left panel) and zooplankton species/taxa (right panel) for all 24 assemblages. For each figure, from a to x, column corresponds to the entire values taken by an environmental variable (e.g. distance to coast or silicate). For each variable (column) all values were divided into 100 categories standardised between 0 and 1, bottom categories (0) corresponding to the smallest values taken by an environmental variable. Colour indices denote the percentage of phytoplankton species/taxa (on left) or zooplankton species/taxa (on right) of an assemblage found into a category. Red colour indicates that the majority of the zooplankton or phytoplankton species/taxa composing the assemblage are found in these environmental categories. Blue colour indicates that no (or a few) zooplankton or phytoplankton species/taxa were found in these environmental categories. Panels are classified from a to x by decreasing taxonomic richness. The number at the top left of each panel corresponds to the assemblage number (see Figure 2 and Figure S4) and the number at the top right (n) indicates the taxonomic richness of each assemblage.


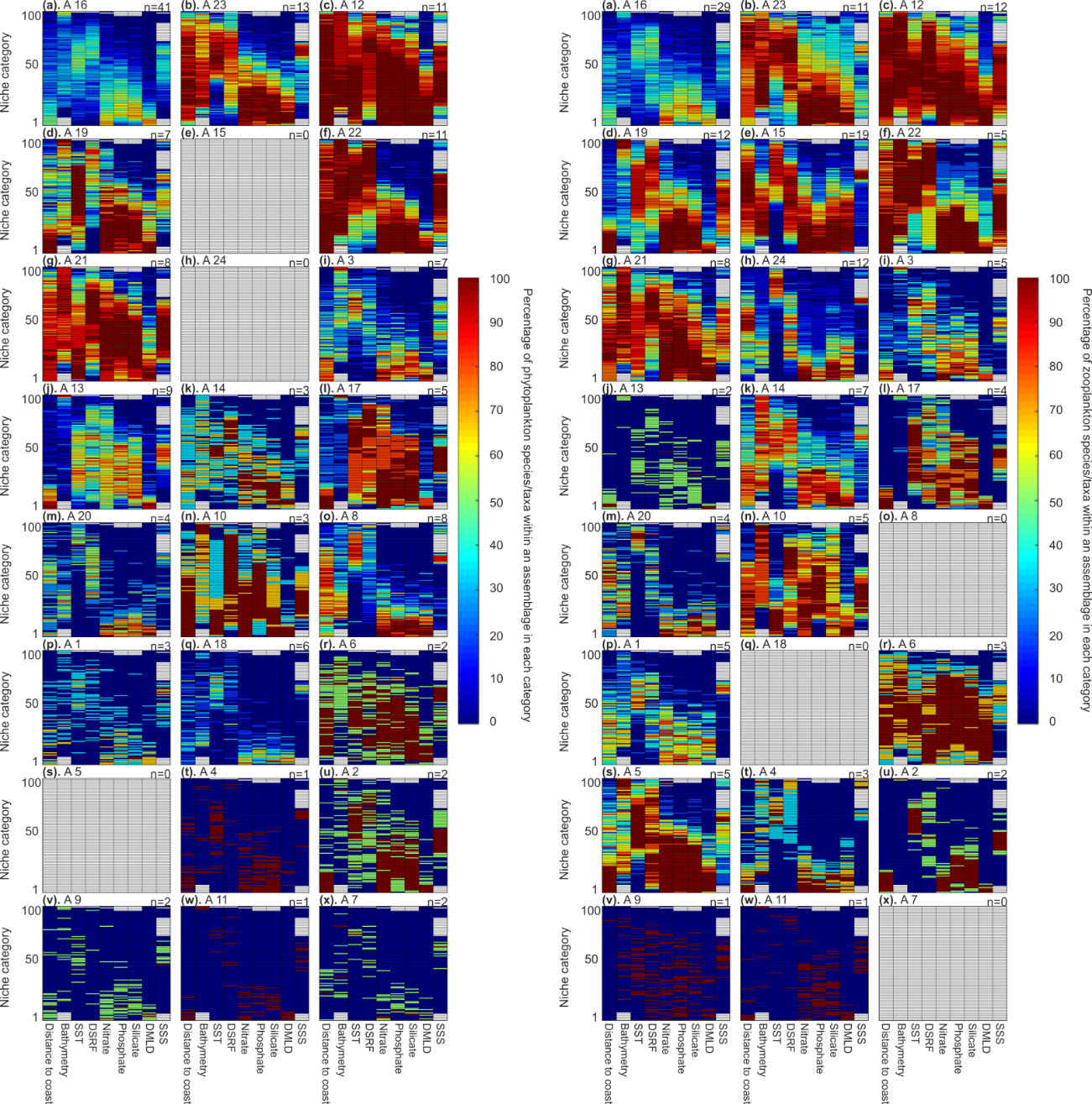


**SUPPLEMENTARY TEXTS**

**Supplementary Text 1**

**CPR limitations**

The Continuous Plankton Recorder (CPR) survey is a long-term plankton-monitoring programme, operated currently by the Marine Biological association of Plymouth. The CPR is the longest and most extensive programme of that kind in the world, started in 1931, with minor modifications to the mechanism after 1948. After a break during World War 2, zooplankton sampling has not been changed since 1948, and phytoplankton since 1958 (2). The machine is a high-speed plankton recorder, towed behind voluntary merchant ships, called “ships of opportunity”, operating at a depth of approximately 7 m, filtering phytoplankton and zooplankton (3).

A sampling road effect may affect phytoplankton abundance data. Usually phytoplankton cells are smaller than zooplankton species so their abundance is greater; this is an expression of Damuth’s law (4), which links body size of an organism to its species population density by an inverse relationship, i.e. smaller organisms tend to have a greater population density.

The CPR survey currently records ~1000 plankton entities (many to species level) in routine taxonomic analysis dating back over multi-decades. Due to the mesh size of CPR silks, many plankton species are only semi-quantitatively sampled owing to the small size of the organisms. In the case of phytoplankton there is thus a bias towards recording larger armoured flagellates and chain-forming diatoms and that smaller species abundance estimates from cell counts will probably be underestimated in relation to other water sampling methods. However, the proportion of the population that is retained by the CPR silk reflects the major changes in abundance, distribution and specific composition, i.e. the percentage retention is roughly constant within each species even with very small-celled species (5). Potential under estimation of zooplankton abundances has recently been thoroughly statistically explored by Hélaouët *et al.,* 2016 (6) who found that while the CPR survey does underestimate abundance in some cases the CPR survey does give a correct picture of both temporal (i.e. seasonal and diel scales) and spatial (i.e. regional to basin-scale) changes in zooplankton taxa in this case the species *Calanus finmarchicus*. The Hélaouët *et al.* study also showed that while the CPR sampling is restricted to the surface waters ~10 m in depth the seasonal and diel patterns of abundance of *C. finmarchicus* were positively correlated to patterns of abundance to a depth of 100 m.

The CPR is not a perfect sampling mechanism by any means and it will underestimate components of the plankton, e.g. large plankton like fish larvae and delicate gelatinous plankton. This has been well documented and users of the data are advised of the CPR's limitations (7). It is also widely recognised that all plankton sampling systems have their own limitations and nuances and all underestimate abundance to some degree and that the varying mechanisms are not always directly comparable (8). However, in the case of krill, as in this study, the CPR may be underestimating the larger species due to the small sampling aperture (12.7 × 12.7 mm) of the CPR mechanism. As estimated in Silva *et al.,* 2014 (9), ~87% of the larvae and juveniles of krill are captured by the CPR but it is less efficient at catching larger krill, therefore, the CPR krill data may not represent absolute abundances of adult krill but will provide indices of the larval and juvenile stages of the three most common euphausiid species in the North Atlantic as well as the adult stages of the smallest species (*T. longicaudata*). It is likely that the large adult stages of the euphausiid species *M. norvegica* are underestimated in CPR sampling. Taking the northern North Atlantic Ocean as a whole, as well as within the specific area of the present study, the most numerically abundant near-surface species is *T. longicaudata*, making it likely that our findings are representative of real changes in euphausiid populations. However, further north, in areas such as the Barents Sea (not covered in this study), where the species *M. norvegica* can dominate, the CPR sampling is less likely to reflect real changes in the adult euphausiid populations. It is not known whether certain taxa such as krill are capable of gear avoidance and in some cases this might be a bias for some fast moving taxa, however, since CPR sampling is conducted at high-speeds (up to 20 knots), gear avoidance is considered to be fairly minimal (8). A detailed study has been conducted on flow rate and ship speed on CPR sampling (10) given that the speed of the ships has, in some circumstances, increased since the 1960s, which may impact sample efficiencies. However, no significant correlation was found between the long-term changes in the speed of the ships and two commonly used indicators of plankton variability: the Phytoplankton Colour and the Total Copepods indices. This absence of relationship may indicate that the effect found is small in comparison with the influence of hydroclimatic forcing (10). For further details on the technical background, methods, consistency, and comparability of CPR sampling, see (11).

It is important to note that the CPR survey is the only scientific monitoring programme of that kind in the world, with no equivalent existing program. It covers an important time scale from 1948 to present, still active, with a large spatial scale covering the whole North Atlantic Ocean and its adjacent seas such as the Channel, the Celtic Sea and the North Sea. It has become “the most extensive long-term survey of marine organisms in the world” (12).

**References**

1. Beaugrand G, Edwards M, Hélaouët P. An ecological partition of the Atlantic Ocean and its adjacent seas. Prog Oceanogr. avr 2019;173:86‑102.

2. Warner AJ, Hays GC. Sampling by the continuous plankton recorder survey. Prog Oceanogr. janv 1994;34(2‑3):237‑56.

3. Hays GC, Warner AJ. Consistency of Towing Speed and Sampling Depth for the Continuous Plankton Recorder. J Mar Biol Assoc U K. nov 1993;73(4):967‑70.

4. Damuth J. Population density and body size in mammals. Nature. avr 1981;290(5808):699‑700.

5. Edwards M, Johns DG, Leterme SC, Svendsen E, Richardson AJ. Regional climate change and harmful algal blooms in the northeast Atlantic. Limnol Oceanogr. mars 2006;51(2):820‑9.

6. Hélaouët P, Beaugrand G, Reygondeau G. Reliability of spatial and temporal patterns of C. finmarchicus inferred from the CPR survey. J Mar Syst. janv 2016;153:18‑24.

7. Richardson AJ, Walne AW, John AWG, Jonas TD, Lindley JA, Sims DW, et al. Using continuous plankton recorder data. Prog Oceanogr. janv 2006;68(1):27‑74.

8. Owens NJP, Hosie GW, Batten SD, Edwards M, Johns DG, Beaugrand G. All plankton sampling systems underestimate abundance: Response to “Continuous plankton recorder underestimates zooplankton abundance” by J.W. Dippner and M. Krause. J Mar Syst. déc 2013;128:240‑2.

9. Silva T, Gislason A, Licandro P, Marteinsdóttir G, Ferreira ASA, Gudmundsson K, et al. Long-term changes of euphausiids in shelf and oceanic habitats southwest, south and southeast of Iceland. J Plankton Res. 1 sept 2014;36(5):1262‑78.

10. Jonas TD, Walne AW, Beaugrand G, Gregory L, Hays GC. The volume of water filtered by a Continuous Plankton Recorder sample: the effect of ship speed. J Plankton Res. 18 août 2004;26(12):1499‑506.

11. Batten SD, Clark R, Flinkman J, Hays G, John E, John AWG, et al. CPR sampling: the technical background, materials and methods, consistency and comparability. Prog Oceanogr. août 2003;58(2‑4):193‑215.

12. Reid PC, Colebrook JM, Matthews JBL, Aiken J, Continuous Plankton Recorder Team. The Continuous Plankton Recorder: concepts and history, from Plankton Indicator to undulating recorders. Prog Oceanogr. août 2003;58(2‑4):117‑73.
